# NLRP12 downregulates the Wnt/**β**-catenin pathway via interaction with STK38 to suppress colorectal cancer

**DOI:** 10.1172/JCI166295

**Published:** 2023-10-02

**Authors:** Shahanshah Khan, Youn-Tae Kwak, Lan Peng, Shuiqing Hu, Brandi L. Cantarel, Cheryl M. Lewis, Yunpeng Gao, Ram S. Mani, Thirumala-Devi Kanneganti, Hasan Zaki

**Affiliations:** 1 Department of Pathology,; 2 Bioinformatics Core Facility, Lyda Hill Department of Bioinformatics, and; 3 Simmons Comprehensive Cancer Center, UT Southwestern Medical Center, Dallas, Texas, USA.; 4 Department of Immunology, St. Jude Children’s Research Hospital, Memphis, Tennessee, USA.

**Keywords:** Gastroenterology, Colorectal cancer, Tumor suppressors

## Abstract

Colorectal cancer (CRC) at advanced stages is rarely curable, underscoring the importance of exploring the mechanism of CRC progression and invasion. NOD-like receptor family member NLRP12 was shown to suppress colorectal tumorigenesis, but the precise mechanism was unknown. Here, we demonstrate that invasive adenocarcinoma development in *Nlrp12*-deficient mice is associated with elevated expression of genes involved in proliferation, matrix degradation, and epithelial-mesenchymal transition. Signaling pathway analysis revealed higher activation of the Wnt/β-catenin pathway, but not NF-κB and MAPK pathways, in the *Nlrp12*-deficient tumors. Using *Nlrp12*–conditional knockout mice, we revealed that NLRP12 downregulates β-catenin activation in intestinal epithelial cells, thereby suppressing colorectal tumorigenesis. Consistent with this, *Nlrp12*-deficient intestinal organoids and CRC cells showed increased proliferation, accompanied by higher activation of β-catenin in vitro. With proteomic studies, we identified STK38 as an interacting partner of NLRP12 involved in the inhibition of phosphorylation of GSK3β, leading to the degradation of β-catenin. Consistently, the expression of NLRP12 was significantly reduced, while p-GSK3β and β-catenin were upregulated in mouse and human colorectal tumor tissues. In summary, NLRP12 is a potent negative regulator of the Wnt/β-catenin pathway, and the NLRP12/STK38/GSK3β signaling axis could be a promising therapeutic target for CRC.

## Graphical abstract

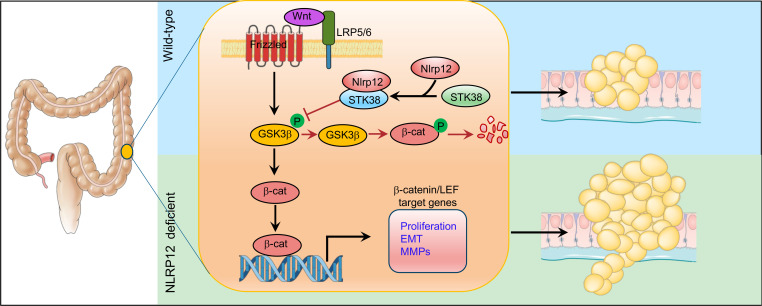

## Introduction

With more than 1.2 million newly diagnosed cases per year, colorectal cancer (CRC) is the third most common cancer and the fourth leading cause of cancer-related death worldwide (1). Major challenges for CRC treatment are cancer metastasis and resistance to conventional chemo- and radiotherapies. Approximately 40% of human CRC progresses to metastasis, and the survival rate for metastatic CRC is below 10% (2). Thus, it is important to understand the molecular mechanism of CRC pathogenesis and identify molecular checkpoints for CRC initiation, progression, and metastasis.

The Wnt/β-catenin signaling pathway, which regulates development, proliferation, stemness, cell-cell adhesion, epithelial-mesenchymal transition (EMT), and other essential physiological functions, is the most significantly altered pathway in clinical CRC specimens (3–5). β-Catenin is activated when Wnt ligands bind to the receptor complex consisting of Frizzled and LRP5/6 proteins, leading to the repression of the β-catenin destruction complex composed of adenomatous polyposis coli (APC), AXIN, and glycogen synthase kinase 3β (GSK3β) (6–8). APC, which sequesters β-catenin in the cytoplasm, is a cancer suppressor, and its mutations have been detected in 80% of sporadic CRC (3, 9). Dysfunction of APC or other signaling adapters leads to the translocation of β-catenin into the nucleus, where it binds to transcription factors T-cell factor (TCF) and lymphoid enhancer factor (LEF), allowing expression of Wnt target genes (10, 11).

In addition to Wnt/β-catenin and other oncogenic pathways, tumor induction, progression, and metastasis can be modulated by multiple extrinsic and intrinsic factors, including diet, gut microbiome, immune response, metabolism, epigenetics, and others (12). NLRP12, a cytosolic pattern recognition receptor in the family of NOD-like receptors (NLRs), has recently emerged as a negative regulator of inflammation and tumorigenesis (13–17). Mice deficient in *Nlrp12* showed increased susceptibility to azoxymethane (AOM) plus dextran sodium sulfate (DSS)–induced colitis and colitis-associated colorectal tumorigenesis (13, 17). We observed that approximately 30% of *Nlrp12^–/–^* mice developed invasive adenocarcinoma, while none of the wild-type (WT) mice showed tumor invasiveness at the same time, suggesting a critical role for NLRP12 in CRC progression and invasion (17). NLRP12 has been characterized as a negative regulator of inflammatory signaling pathways, including NF-κB and MAPK (13, 16, 17). NLRP12 also modulates gut microbiota composition, influencing colitis susceptibility (18). In contrast, a recent study suggests that NLRP12 promotes inflammation and inflammatory cell death, PANoptosis, during hemolytic disease (19). However, the precise mechanism through which NLRP12 suppresses CRC promotion, invasion, and migration remains unknown.

Here, we mechanistically defined the role of NLRP12 in CRC progression and invasion. Our data suggest that increased colorectal tumorigenesis and invasiveness of *Nlrp12^–/–^* mice are independent of altered gut microbiota composition, but associated with higher expression of protooncogenes, matrix metalloproteinases (MMPs), and EMT markers. We showed that NLRP12 intrinsically regulates these genes by suppressing the Wnt/β-catenin pathway in intestinal epithelial cells. With biochemical and proteomic studies, we explored the molecular mechanism of NLRP12-mediated regulation of the Wnt/β-catenin pathway and demonstrated that NLRP12 phosphorylates GSK3β via interaction with STK38, leading to the degradation of β-catenin. Overall, this study establishes an intestinal epithelial cell–specific function of NLRP12 that potentially inhibits colorectal tumor progression and invasion.

## Results

### NLRP12 suppresses genes involved in proliferation, matrix degradation, and EMT during colorectal tumorigenesis.

**Figure 1:**
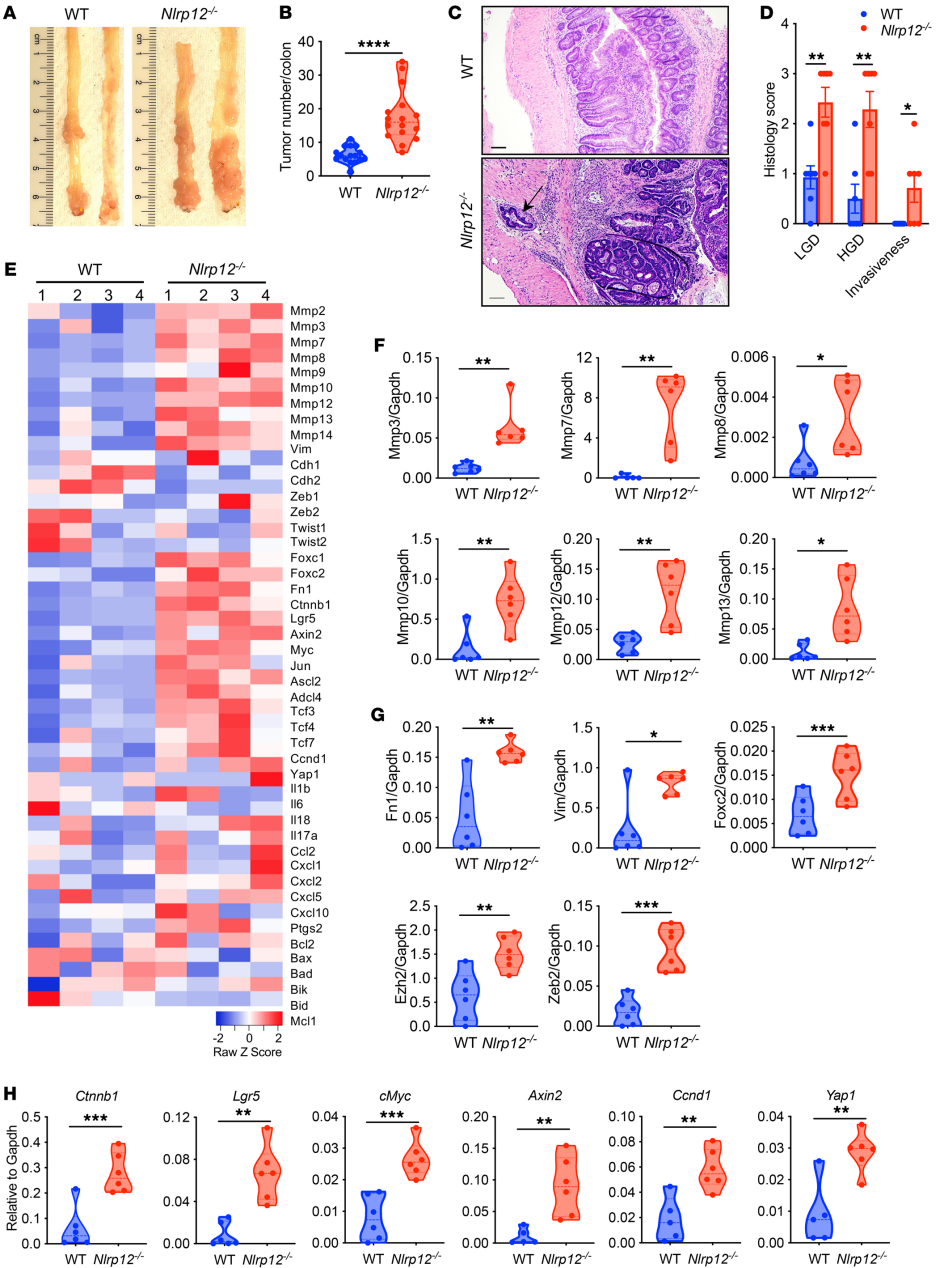
NLRP12 suppresses invasive adenocarcinoma and downregulates genes involved in proliferation, epithelial-mesenchymal transition (EMT), and invasion. Colorectal tumorigenesis was induced in WT and *Nlrp12^–/–^* mice (*n* = 16/group) using the AOM plus DSS regimen. (**A** and **B**) Mice were sacrificed 12 weeks after AOM, and tumor burden was counted. (**C** and **D**) Tumor-bearing colon sections (*n* = 7) were stained with H&E and examined histopathologically. (**C**) Representative images of H&E-stained colons. The arrow indicates the invasion of the tumor into the stroma. Scale bars: 100 μm. (**D**) Semiquantitative scoring for low-grade dysplasia (LGD), high-grade dysplasia (HGD), and invasive adenocarcinoma. Data represent mean ± SEM. Experiments were repeated at least 3 times, and data from a representative experiment are presented. (**E**) RNA isolated from tumors was used for RNA-seq analysis. The heatmap shows the expression of selected genes. (**F**–**H**) The expression of MMPs (**F**), EMT markers (**G**), and Wnt target genes (**H**) in the tumor was measured by real-time RT-PCR. Data represent mean ± SEM. Experiments were repeated 2 times, and data from a representative experiment are presented. *P < 0.05, **P < 0.01, ***P < 0.001, ****P < 0.0001 by unpaired, 2-tailed Student’s *t* test.

We previously demonstrated that *Nlrp12*-deficient mice are susceptible to chemical carcinogen–induced colorectal tumorigenesis, with a higher incidence of high-grade dysplasia and invasive adenocarcinoma (17). Invasion from its primary site into the stroma is the first step of cancer metastasis, a condition that accounts for the mortality of cancer patients (20). We, therefore, wondered whether and how NLRP12 suppresses cancer invasiveness. To address this concern, we wanted to identify transcriptomic signatures of invasiveness regulated by NLRP12. Hence, we induced colorectal tumorigenesis in WT and *Nlrp12^–/–^* mice with the administration of AOM plus DSS (17, 21). Consistent with the previous observation, there was a higher tumor burden and higher incidence of high-grade dysplasia and invasive adenocarcinoma (Figure 1, A–D); approximately 50% of *Nlrp12^–/–^* mice exhibited invasive adenocarcinoma, while no tumor invasiveness was observed in WT mice (Figure 1, C and D). The gene expression profile in WT and *Nlrp12^–/–^* tumors was measured by RNA-seq. Interestingly, the MMPs, including *Mmp7*, -*8*, -*10*, and -*12*, were highly represented in the top 100 upregulated genes in *Nlrp12^–/–^* tumors compared with WT tumors (Figure 1E and Supplemental Figure 1A; supplemental material available online with this article; https://doi.org/10.1172/JCI166295DS1). MMPs degrade the extracellular matrix of the basement membrane, which provides physical support to the tumor, facilitating EMT, invasion, and metastasis of the tumor (22). In addition to MMPs, there was higher expression of *Foxc2*, a transcription factor associated with EMT and cancer metastasis (23), and stemness marker *Lgr5* (Supplemental Figure 1A and Supplemental Table 2). To obtain further insights, we analyzed the expression of some known genes involved in EMT, proliferation, oncogenesis, cell death, and inflammation (9, 24, 25). *Nlrp12^–/–^* tumors exhibited significantly higher levels of *Mmp2*, -*3*, -*7*, -*8*, -*9*, -*10*, -*12* and -*13*, and EMT-associated genes, including *Fn1*, *Vim*, *Foxc2*, *Zeb1*, and *Zeb2* (Figure 1E and Supplemental Figure 1B). In comparison with WT tumors, the expression of many proproliferative genes and oncogenes, including *Myc*, *Ccnd1*, *Lgr5*, *Ctnnb1*, and *Jun*, was higher in *Nlrp12^–/–^* tumors (Figure 1E and Supplemental Figure 1B). A major pathway that regulates these oncogenes is Wnt/β-catenin. Consistently, there was significantly higher expression of other Wnt target genes, including *Axin2*, *Ascl2*, *Tcf3*, *Tcf4*, and *Yap1* in *Nlrp12^–/–^* tumors (Figure 1E and Supplemental Figure 1B). Notably, no significant difference was observed in the expression of proinflammatory cytokines and chemokines between WT and *Nlrp12^–/–^* tumors (Figure 1E).

To validate the RNA-seq data, we measured the expression of MMPs, EMT markers, and Wnt target genes by real-time RT-PCR. Consistent with the RNA-seq data, there was higher expression of *Mmp3*, -*7*, -*8*, -*10*, -*12*, and -*13* (Figure 1F), EMT markers *Vim*, *Fn1*, *Foxc2*, *Ezh2*, and *Zeb2* (Figure 1G), and Wnt target genes *Ctnnb1*, *Lgr5*, *cMyc*, *Axin2*, *Ccnd1*, and *Yap1* in *Nlrp12^–/–^* tumors (Figure 1H). We also measured the expression of *Il1b*, *Il6*, *Cxcl1*, *Cxcl2*, and *Ifng* but could not see any difference between WT and *Nlrp12^–/–^* tumors (Supplemental Figure 1C), supporting the RNA-seq data (Figure 1E). Overall, these results suggest that higher tumorigenesis and invasiveness in *Nlrp12^–/–^* mice are associated with higher expression of genes related to oncogenesis, EMT, and metastasis.

### CRC susceptibility of Nlrp12-deficient mice is independent of gut microbiota composition.

**Figure 2:**
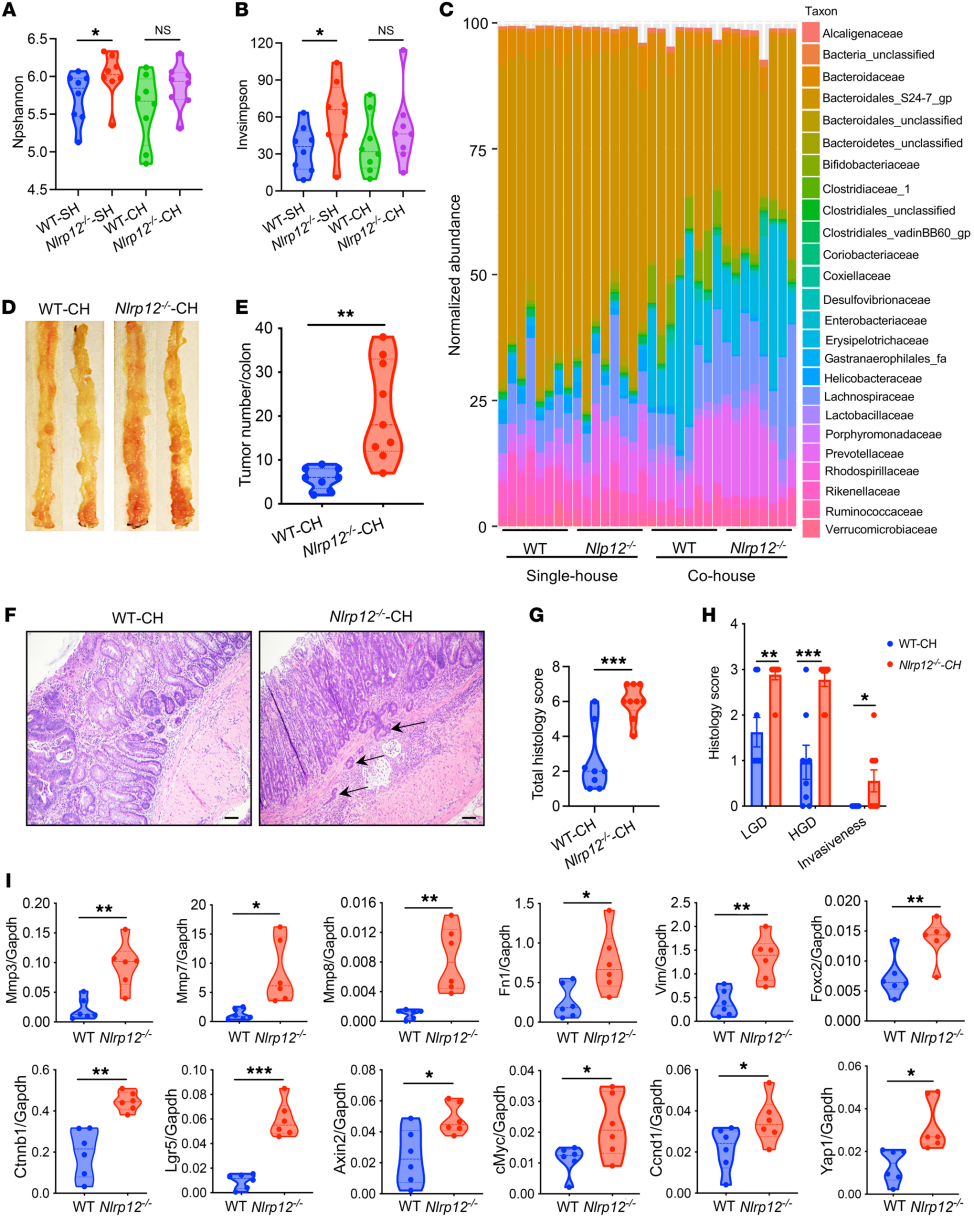
CRC susceptibility of *Nlrp12^–/–^* mice is independent of gut microbiota composition. WT and *Nlrp12^–/–^* mice (*n* = 8–9/group) were cohoused for 4 weeks. (**A**–**C**) Fecal DNA was isolated from separately housed (SH) or cohoused (CH) WT and *Nlrp12^–/–^* mice and sequenced for the 16S rRNA gene. (**A** and **B**) α-Diversity showing species richness was analyzed by nonparametric Shannon (npShannon) index (**A**) and Inverse Simpson index (**B**). Data represent mean ± SEM. (**C**) Stack bar plot showing the relative distribution of the microbial community at family levels. (**D**–**H**) Colorectal tumorigenesis was induced in cohoused WT and *Nlrp12^–/–^* mice with AOM plus DSS. (**D** and **E**) Mice were sacrificed 12 weeks after AOM, and tumor burden was counted. (**F**) Representative images of H&E-stained colons. The arrow indicates the invasion of colorectal tumor into the stroma. Scale bars: 100 μm. (**G** and **H**) Semiquantitative histopathological scorings for low-grade dysplasia (LGD), high-grade dysplasia (HGD), and invasiveness. (**I**) RNA isolated from tumors was used to measure the expression of the indicated genes by real-time RT-PCR. Data represent mean ± SEM. *P < 0.05, **P < 0.01, ***P < 0.001 by Mann-Whitney test (**A** and **B**) or unpaired, 2-tailed Student’s *t* test (**E** and **G**–**I**). Experiments were repeated 2 times, and data from a representative experiment are presented.

Gut microbiota plays an important role in CRC pathogenesis (26). Previous studies reported that *Nlrp12^–/–^* mice harbored altered gut microbiota composition (18). Our analysis of gut microbial 16S rRNA also showed higher species richness (α-diversity) in *Nlrp12^–/–^* mice compared with WT mice (Figure 2, A and B). To understand whether higher colorectal tumorigenesis and invasiveness were due to the altered microbiota composition of *Nlrp12^–/–^* mice, we cohoused WT and *Nlrp12^–/–^* mice for 4 weeks. Cohousing helped equalize the gut microbiota of WT and *Nlrp12^–/–^* mice, as evidenced by the species richness and relative abundance of different bacterial families (Figure 2, A–C). We then induced colorectal tumorigenesis with the AOM/DSS regimen in cohoused WT and *Nlrp12^–/–^* mice. Consistent with the data obtained from separately housed mice (Figure 1, A and B), cohoused *Nlrp12^–/–^* mice developed a significantly higher number of tumors compared with WT (Figure 2, D and E). Histopathological analysis showed that tumors of *Nlrp12^–/–^* mice were mostly high-grade dysplasia, with several showing invasion into the stroma (Figure 2, F–H). Furthermore, the expression of EMT genes and oncogenes, including *Mmp3*, *Mmp7*, *Mmp8*, *Fn1*, *Vim*, *Foxc2*, *Ctnnb1*, *Lgr5*, *Axin2*, *cMyc*, *Ccnd1*, and *Yap1*, was significantly high in the tumor of cohoused *Nlrp12^–/–^* compared with WT mice (Figure 2I). Littermate *Nlrp12^–/–^* mice also developed higher tumor burden and invasive adenocarcinoma compared with littermate WT mice (Supplemental Figure 2, A–C). Oncogenes, EMT markers, and metastatic genes were highly expressed in tumors of littermate *Nlrp12^–/–^* mice (Supplemental Figure 2D).

To further examine whether the gut microbiota of *Nlrp12^–/–^* mice is tumor promoting, we performed a fecal transplantation study in which fecal microbiota from WT or *Nlrp12^–/–^* mice were transplanted into germ-free mice. Two weeks following fecal transplantation, mice were treated with AOM plus 3 cycles of DSS. Colitis and tumorigenesis in both fecal transplanted groups were comparable (Supplemental Figure 2, E–I). Together, these data implied that altered gut microbiota composition is not responsible for higher CRC susceptibility of *Nlrp12*-deficient mice.

### Increased invasive adenocarcinoma development of Nlrp12^–/–^ mice is associated with higher activation of the Wnt/β-catenin pathway.

**Figure 3:**
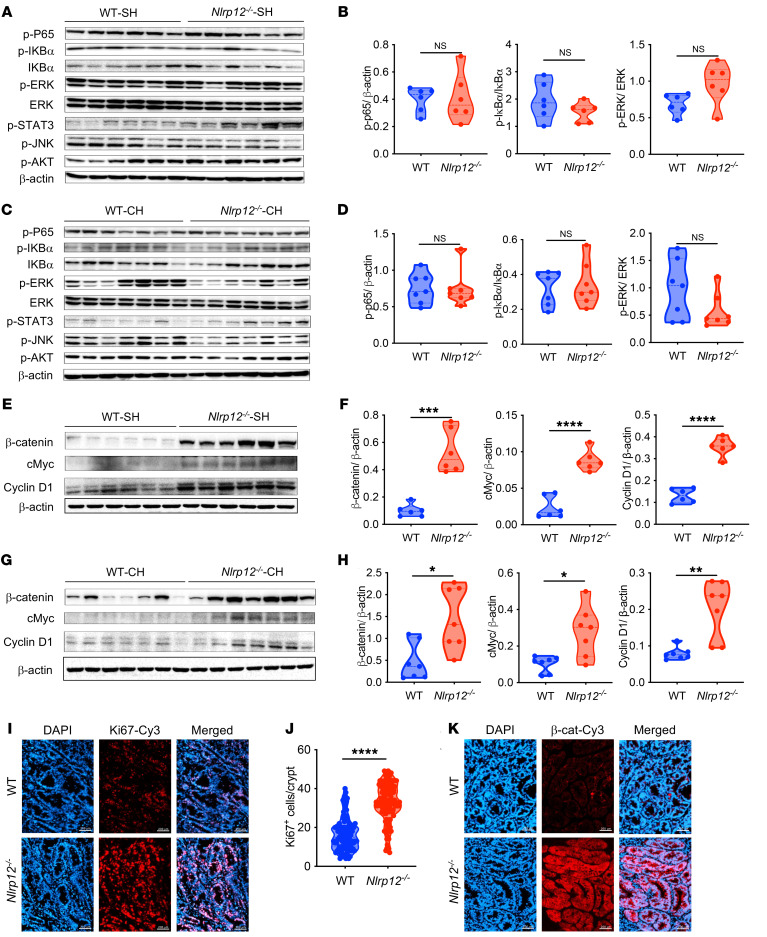
Increased tumorigenesis of *Nlrp12^–/–^* mice is associated with higher β-catenin activation in the tumor. WT and *Nlrp12^–/–^* mice were separately housed (SH) (*n* = 6/group) or cohoused (CH) (*n* = 7/group). Colorectal tumorigenesis was induced by AOM plus DSS treatment. Mice were sacrificed 12 weeks after AOM injection. (**A**–**D**) Tumors were analyzed for the activation of indicated signaling pathways by Western blotting. Band intensities of p-P65, p-IκBa, and p-ERK were measured densitometrically. Data represent mean ± SEM. (**E**–**H**) The expression of β-catenin, cMyc, and cyclin D1 in tumors was analyzed by Western blotting. Band intensities of β-catenin, cMyc, and cyclin D1 were measured densitometrically. Data represent mean ± SEM. (**I** and **J**) Colon sections were stained for Ki67 and positive cells (red) were counted under 20× objective (*n* = 3 mice/group). Scale bars: 200 μm. Data represent mean ± SEM. (**K**) Tumor-bearing colon sections were immunostained for β-catenin (red). The nuclei were stained with DAPI (blue). Scale bars: 200 μm. All experiments were repeated at least 2 times, and data from a representative experiment are presented. *P < 0.05, **P < 0.01, ***P < 0.001, ****P < 0.0001 by unpaired, 2-tailed Student’s *t* test.

The above observations led us to investigate the underlying mechanism of upregulated expression of oncogenes and EMT markers in *Nlrp12^–/–^* tumors. We previously showed that increased colitis susceptibility of *Nlrp12^–/–^* mice is associated with higher activation of NF-κB and ERK pathways (17). Here, we confirmed our earlier findings that *Nlrp12^–/–^* mice develop more severe colitis with AOM/DSS treatment (Supplemental Figure 3, A–D), which is accompanied by increased activation of NF-κB and ERK, and higher expression of inflammatory cytokines, including *Il6*, *Il1b*, *Cxcl1*, and *Cxcl2*, at the early time points of colorectal tumorigenesis (Supplemental Figure 3, E–G). To understand whether these and other inflammatory pathways are dysregulated in *Nlrp12^–/–^* colorectal tumors, we measured the activation of NF-κB, ERK, JNK, STAT3, and AKT in tumors collected on day 80 after AOM/DSS treatment. Surprisingly, there was no major difference in the activation of NF-κB and MAPK pathways between WT and *Nlrp12^–/–^* mice housed separately or together (Figure 3, A–D).

Since our RNA-seq data show higher induction of Wnt target genes in *Nlrp12^–/–^* tumors, we wondered whether the Wnt/β-catenin pathway is dysregulated. Interestingly, the protein levels of β-catenin and its downstream targets cMyc and cyclin D1 were markedly higher in *Nlrp12^–/–^*tumors compared with those of WT tumors (Figure 3, E and F). β-Catenin, cMyc, and cyclin D1 remained high in tumors of *Nlrp12^–/–^* mice even after cohousing with WT (Figure 3, G and H). Corroborating the role of cMyc and cyclin D1 in proliferation, there was increased proliferation as measured by Ki67 staining in *Nlrp12^–/–^* tumors (Figure 3, I and J). Increased activation of the Wnt/β-catenin pathway was further confirmed by immunostaining of β-catenin, showing that β-catenin levels were markedly higher in *Nlrp12^–/–^* tumors (Figure 3K). These data suggest a role for NLRP12 in the suppression of the Wnt/β-catenin pathway during colorectal tumorigenesis.

### Nlrp12 deficiency leads to higher tumor development in Apc^min/+^ mice.

**Figure 4:**
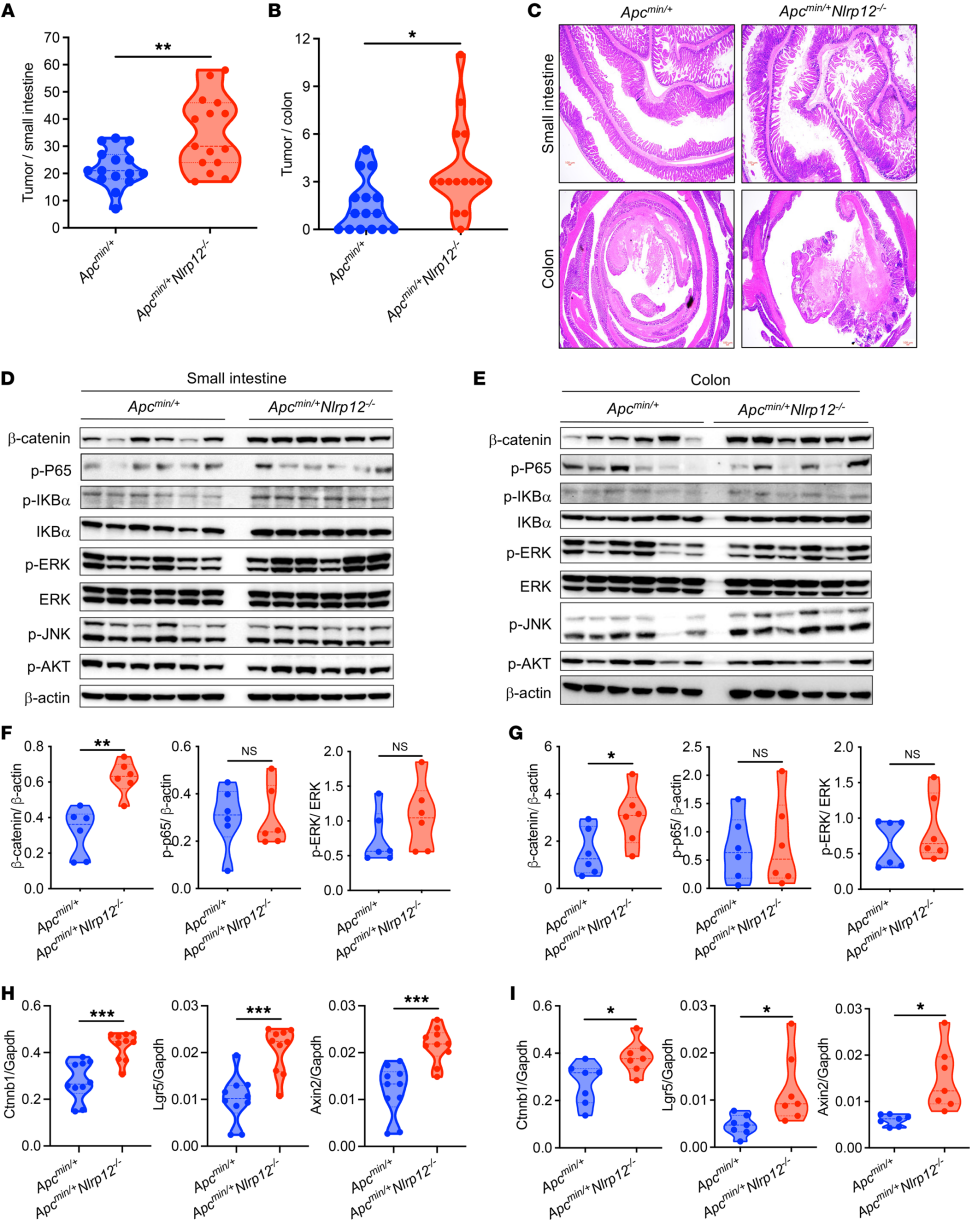
*Nlrp12* deficiency promotes colorectal tumorigenesis in *Apc^min/+^* mice. *Apc^min/+^* and *Apc^min/+^* *Nlrp12^–/–^* mice (*n* = 15/group) were sacrificed 5 months after birth, and the number of tumors was counted in the small (**A**) and large intestine (colon) (**B**). (**C**) Representative images of H&E-stained small intestine and colons at low (2×) magnifications. Scale bars: 100 μm. (**D**–**G**) Homogenates from tumor-bearing small intestines and colons were analyzed for the activation of β-catenin, p-P65, p-IκBα, IκBα, p-ERK, ERK, p-JNK, p-AKT, and β-actin by Western blotting. Band intensities of p-P65, p-ERK, and β-catenin relative to β-actin were measured. Data represent mean ± SEM. (**H** and **I**) RNA isolated from small intestine and tumor-bearing colons was measured for the expression of indicated genes by real-time RT-PCR. Data represent mean ± SEM. Experiments were repeated at least 3 times, and data from a representative experiment are presented. *P < 0.05, **P < 0.01, ***P < 0.001 by unpaired, 2-tailed Student’s *t* test.

The homeostatic level of Wnt/β-catenin activation is maintained by the β-catenin destruction complex. APC is a core component of the destruction complex that holds β-catenin in the cytoplasm, allowing its phosphorylation and proteasomal degradation (27). Heterozygous mice carrying a mutation in *Apc* (*Apc^min/+^*) in intestinal epithelial cells spontaneously develop multiple intestinal neoplasia (min) (28). To verify the role of NLRP12 in a genetic model of CRC, we crossed *Nlrp12^–/–^* mice with *Apc^min/+^* mice. There was an increased burden of polyps in small intestines and colons of *Apc^min/+^* *Nlrp12^–/–^* mice (Figure 4, A–C). As we analyzed signaling pathways in small and large intestinal tissues from *Apc^min/+^* and *Apc^min/+^* *Nlrp12^–/–^* mice, we observed strikingly higher levels of β-catenin in small intestines and colons of *Apc^min/+^* *Nlrp12^–/–^* mice, while no remarkable difference was observed in NF-κB, ERK, JNK, and AKT pathways between *Apc^min/+^* and *Apc^min/+^* *Nlrp12^–/–^* mice (Figure 4, D–G). Signaling pathway analysis in tumors from small intestines also revealed higher activation of β-catenin but not NF-κB and MAPK pathways in *APC^min/+^* *Nlrp12^–/–^* mice (Supplemental Figure 4, A and B). Immunostaining of β-catenin further showed increased levels of β-catenin in intestinal polyps of *Apc^min/+^* *Nlrp12^–/–^* compared with *Apc^min/+^* mice (Supplemental Figure 4C). Consistent with the β-catenin level, there was higher expression of Wnt target genes such as *Ctnnb1*, *Lgr5*, and *Axin2* in small intestines and colons of *Apc^min/+^* *Nlrp12^–/–^* relative to *Apc^min/+^* mice (Figure 4, H and I). Altogether, these data demonstrate that NLRP12 deficiency promotes the activation of the Wnt/β-catenin pathway in the tumor epithelium**.**

### Intestinal epithelial cell–specific NLRP12 protects mice against colorectal tumorigenesis.

Next, we wanted to identify cell types showing increased β-catenin activation in *Nlrp12^–/–^* tumors. Hence, we isolated CD45^+^ immune cells and Ep-CAM^+^ epithelial cells from tumors of WT and *Nlrp12^–/–^* mice (Supplemental Figure 5, A and B). As we measured β-catenin levels in isolated cells, we found higher activation of β-catenin and induction of Wnt target genes, including *Ctnnb1*, c*Myc*, and *Ccnd1*, in *Nlrp12^–/–^* tumor epithelial cells (Supplemental Figure 5, C–E). No β-catenin–immunoreactive band was detected in CD45^+^ immune cells, and the expression of *Ctnnb1*, *cMyc*, and *Ccnd1* was comparable between WT and *Nlrp12^–/–^* CD45^+^ cells (Supplemental Figure 5, C and E). No difference in β-catenin activation was also observed in WT and *Nlrp12^–/–^* bone marrow–derived macrophages (BMDMs) when they were stimulated with Wnt3a (Supplemental Figure 5, F and G), while *Nlrp12^–/–^* BMDMs were hyperresponsive to LPS-induced activation of NF-κB and MAPK pathways (Supplemental Figure 5H). These data suggest that NLRP12 regulates the Wnt/β-catenin pathway in epithelial, but not myeloid, cells.

**Figure 5:**
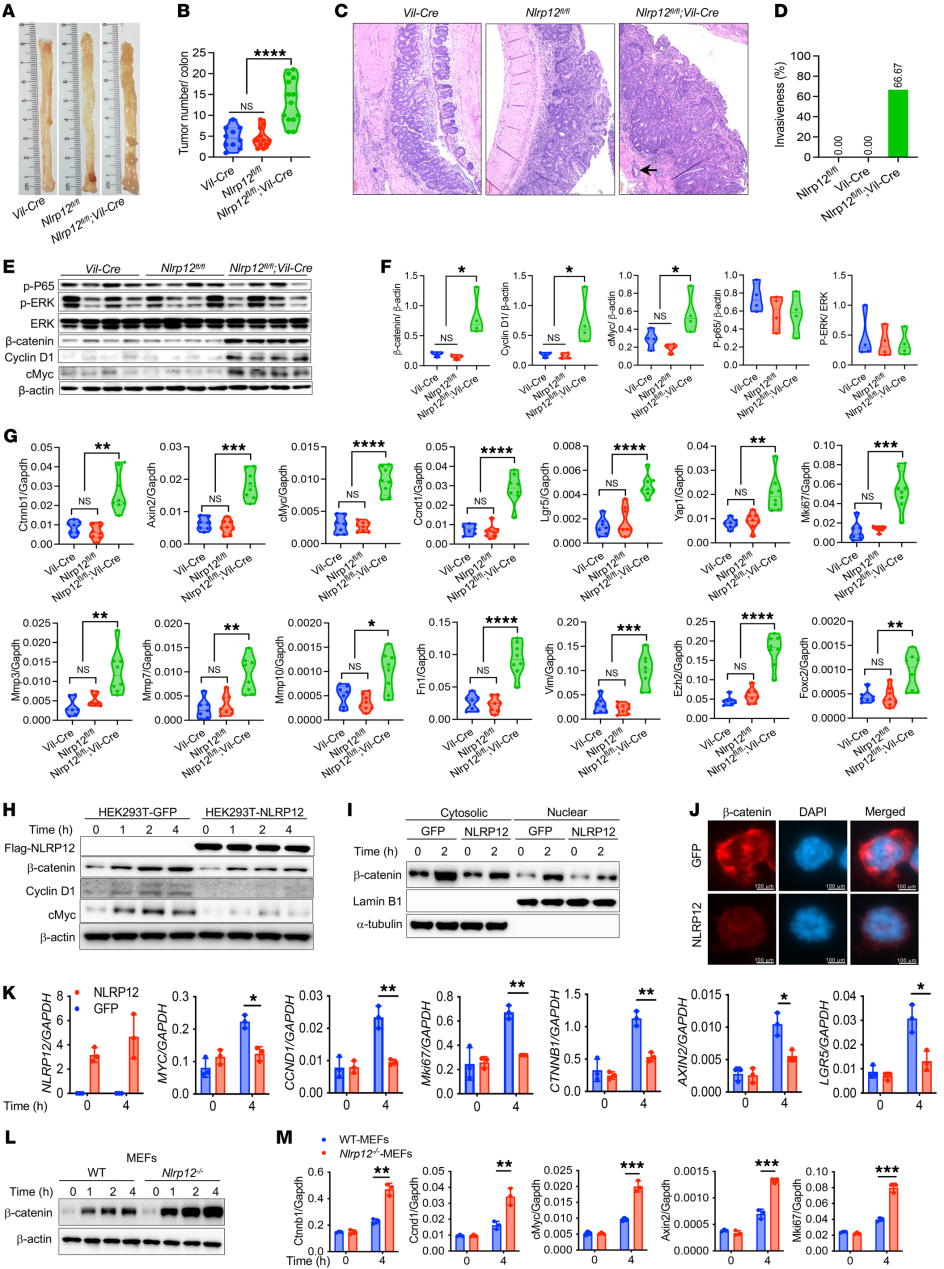
Intestinal epithelial cell–specific NLRP12 suppresses β-catenin activation and colorectal tumorigenesis. (**A**–**G**) *Vil*-Cre (*n* = 10), *Nlrp12^fl/fl^* (*n* = 12), and *Nlrp12^fl/fl^*;*Vil*-Cre (*n* = 13) mice were treated with AOM plus DSS to induce colorectal tumorigenesis. (**A** and **B**) Tumor burden at 12 weeks after AOM/DSS treatment was counted. (**C**) Representative H&E-stained images of colorectal tumors. Scale bars: 100 μm. The arrow indicates tumor invasion into the stroma. (**D**) Percentage of mice showing invasive colorectal adenocarcinoma. (**E**) Tumor homogenates were analyzed for p-P65, p-ERK, ERK, β-catenin, cyclin D1, cMyc, and β-actin by Western blotting. (**F**) Densitometric analysis of band intensity of β-catenin, cyclin D1, cMyc, p-P65, and p-ERK. (**G**) The expression of indicated genes was measured by real-time RT-PCR. Data represent mean ± SEM. Experiments were repeated at least 2 times, and data from a representative experiment are presented. (**H**–**K**) HEK293T cells overexpressing GFP or NLRP12 were stimulated with Wnt3a. (**H**) Cell lysates were analyzed for NLRP12, β-catenin, cMyc, cyclin D1, and β-actin. (**I**) The localization of β-catenin was analyzed by Western blotting of β-catenin in cytoplasmic and nuclear fractions. (**J**) Nuclear localization of β-catenin (red) was visualized microscopically. Scale bars: 100 μm. (**K**) The expression of indicated Wnt target genes was measured by real-time RT-PCR. Data represent mean ± SD of triplicate wells. (**L** and **M**) Mouse embryonic fibroblasts (MEFs) from WT and *Nlrp12^–/–^* mice were cultured and stimulated with Wnt3a. (**L**) The activation of β-catenin was measured by Western blotting. (**M**) The expression of Wnt target genes, including *Ctnnb1*, *Ccnd1*, *cMyc*, *Axin2*, and *MKi67*, was measured by real-time RT-PCR. Data represent mean ± SD of triplicate wells. Experiments were repeated 2 times, and data from a representative experiment are presented. *P < 0.05, **P < 0.01, ***P < 0.001, ****P < 0.0001 by 1-way ANOVA (**B**, **D**, **F**, and **G**) or unpaired, 2-tailed Student’s *t* test (**K** and **M**).

To verify the epithelial cell–specific role of NLRP12 in the regulation of CRC, we generated epithelial cell–specific *Nlrp12*-knockout (*Nlrp12*-KO) mice by crossing *Nlrp12^fl/fl^* mice (Supplemental Figure 6, A and B) with *Vil*-Cre mice (Supplemental Figure 6C). Colorectal tumors were induced with AOM/DSS in *Vil*-Cre, *Nlrp12^fl/fl^*, and *Nlrp12^fl/fl^*;*Vil*-Cre mice. Appreciably, tumor burden in *Nlrp12^fl/fl^*;*Vil*-Cre mice was significantly higher compared with *Vil*-Cre and *Nlrp1^fl/fl^* mice (Figure 5, A and B). Approximately 70% of the *Nlrp12^fl/fl^*;*Vil*-Cre mice exhibited invasive adenocarcinoma development, while no tumor invasion was evident in *Vil*-Cre and *Nlrp12^fl/fl^* mice (Figure 5, C and D). Consistent with whole-body *Nlrp12*-KO mice, intestinal epithelial cell–specific *Nlrp12*-KO mice showed higher activation of β-catenin and expression of its downstream target cMyc and cyclin D1 in colorectal tumors (Figure 5, E and F). With real-time RT-PCR analysis, we confirmed that the expression of *cMyc* and *Ccnd1* and other Wnt target genes, MMPs, and EMT markers was significantly upregulated in *Nlrp12^fl/fl^*;*Vil*-Cre mouse tumors (Figure 5G). Notably, the activation of NF-κB and ERK pathways as well as induction of proinflammatory cytokines, including *Il1b*, *Il6*, and *Tnfa* were comparable in tumors of *Nlrp12*–conditional KO mice and their WT counterparts (Figure 5, E and F, and Supplemental Figure 6D), further emphasizing epithelial cell–specific regulation of the Wnt/β-catenin pathway in NLRP12-mediated protection against CRC.

### NLRP12 is an intrinsic regulator of the Wnt/β-catenin pathway.

To understand whether NLRP12 intrinsically regulates the Wnt/β-catenin pathway, we overexpressed NLRP12 in HEK293T cells and stimulated them with Wnt3a. There was reduced β-catenin activation as well as lowered expression of cyclin D1 and cMyc in HEK293T cells overexpressing NLRP12 (Figure 5H). Such a suppression of β-catenin activation was not observed when NLRP3 or NLRP6 was overexpressed (Supplemental Figure 7A). Wnt3a stimulation leads to the translocation of β-catenin to the nucleus, leading to the induction of Wnt target genes. As such, there were reduced levels of β-catenin in the nucleus of NLRP12-overexpressing HEK293T cells (Figure 5, I and J). Consistent with reduced β-catenin activation, the expression of Wnt target genes, including *CTNNB1*, *AXIN2*, *MYC*, *CCND1*, *Mki67*, and *LGR5*, was markedly lower in NLRP12-overexpressing HEK293T cells (Figure 5K). Since NLRP12 is a known negative regulator of the NF-κB pathway (13–17), we wanted to clarify whether NLRP12-mediated regulation of β-catenin depends on its regulation of the NF-κB pathway. Hence, we stimulated NLRP12-overexpressing HEK293T cells with Wnt3a in the presence or absence of NF-κB inhibitor Sc514. However, inhibition of NF-κB did not affect Wnt3a-mediated activation of β-catenin (Supplemental Figure 7B), implying that NLRP12-mediated regulation of β-catenin is independent of its regulation of the NF-κB pathway.

To examine the function of NLRP12 in regulating the Wnt/β-catenin pathway in primary cells, we cultured mouse embryonic fibroblasts (MEFs) from WT and *Nlrp12^–/–^* mice and stimulated them with Wnt3a. As expected, there was higher β-catenin activation in *Nlrp12^–/–^* MEFs as compared with WT (Figure 5L). Consistently, the Wnt pathway downstream genes, including *Ctnnb1*, *Ccnd1*, *cMyc*, *Axin2*, and *Mki67*, were highly expressed in *Nlrp12^–/–^* MEFs (Figure 5M). These data provide solid evidence that NLRP12 is a potent and intrinsic regulator of the Wnt/β-catenin pathway.

### NLRP12 deficiency promotes the proliferation and migration of cancer cells.

**Figure 6:**
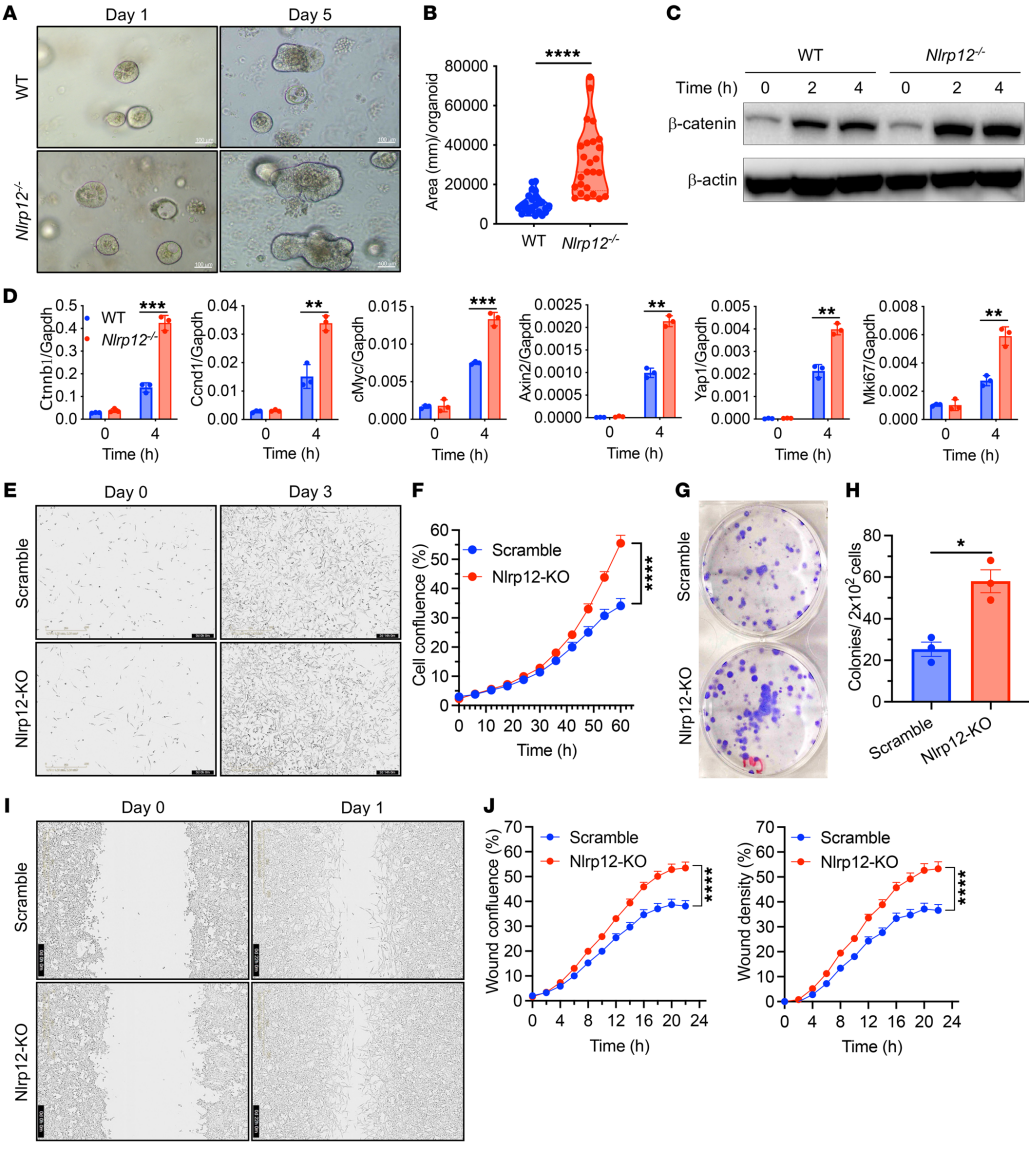
NLRP12 regulates the Wnt/β-catenin pathway and controls proliferation and migration of cancer cells. (**A**–**D**) Crypts from the small intestine of WT and *Nlrp12^–/–^* mice were cultured in a 3D organoid culture system. (**A** and **B**) Organoid growth was monitored, and organoid sizes were measured. (**C**) Organoids were stimulated with Wnt3a, and the activation of β-catenin was measured by Western blotting. (**D**) RNA isolated from Wnt3a-stimulated organoids was measured for the expression of *Ctnnb1*, *Ccnd1*, *cMyc*, *Axin2*, *Yap1*, and *MKi67*. Data represent mean ± SD. Experiments were repeated 3 times, and data of a representative experiment are presented. (**E**–**J**) *Nlrp12*-knockout (*Nlrp12*-KO) MC38 cells were generated with CRISPR/Cas9. (**E** and **F**) Control and *Nlrp12*-KO MC38 cells were cultured, and cell proliferation was monitored by IncuCyte. (**G** and **H**) MC38 and its *Nlrp12*-KO clones were cultured, and colony formation was measured by clonogenic assay. (**I** and **J**) Wound healing of MC38 and *Nlrp12*-KO MC38 cells was monitored by IncuCyte. (**I**) Representative images showing wound on cultured cells (left panel), and migration and cell confluence in wound area (right panel). Data represent mean ± SD of 8 wells (**F**) and 24 wells (**J**). *P < 0.05; **P < 0.01; ***P < 0.001; ****P < 0.0001 by unpaired, 2-tailed Student’s *t* test (**B**, **D**, and **H**) or multiple *t* test (**F** and **J**). Experiments were repeated 3 times, and data of a representative experiment are presented. Scale bars: 100 μm (**A**), 400 μm (**E**), and 600 μm (**I**).

The Wnt/β-catenin pathway plays essential roles in many biological processes and the regulation of cancer (5, 9). To understand the physiological impact of NLRP12-mediated regulation of the Wnt/β-catenin pathway in intestinal stem cell function, we cultured intestinal crypts of WT and *Nlrp12^–/–^* mice in a 3D organoid culture system. *Nlrp12^–/–^* organoids were significantly larger than WT organoids (Figure 6, A and B), indicating a higher proliferation of intestinal stem cells. The rapid proliferation of *Nlrp12^–/–^* organoids was accompanied by higher activation of β-catenin and the expression of Wnt target genes, including *Ctnnb1*, *Ccnd1*, *cMyc*, *Axin2*, *Yap1*, and *Mki67* (Figure 6, C and D).

We next wanted to examine the effect of NLRP12 on cancer cell proliferation and migration. Toward this goal, we knocked out *Nlrp12* in MC38 mouse CRC cells with CRISPR/Cas9 (Supplemental Figure 8). Scrambled or *Nlrp12*-KO MC38 cells were cultured, and their real-time growth was monitored by IncuCyte. Knocking out *Nlrp12* led to increased proliferation of MC38 cells (Figure 6, E and F). Similarly, the clonogenic assay showed increased colony formation by *Nlrp12*-KO MC38 cells (Figure 6, G and H). Since the Wnt/β-catenin pathway regulates genes involved in the invasion and migration of cancer cells, we quantitatively measured the role of NLRP12 in the migration capacity of MC38 cells. *Nlrp12*-KO MC38 cells exhibited higher migration efficiency, as evidenced by the confluence and density of cells in the wound area relative to MC38 cells expressing *Nlrp12* (Figure 6, I and J). These observations support the critical role NLRP12 plays in regulating the proliferation and migration of cancer cells.

### NLRP12 facilitates proteasomal degradation of β-catenin.

**Figure 7:**
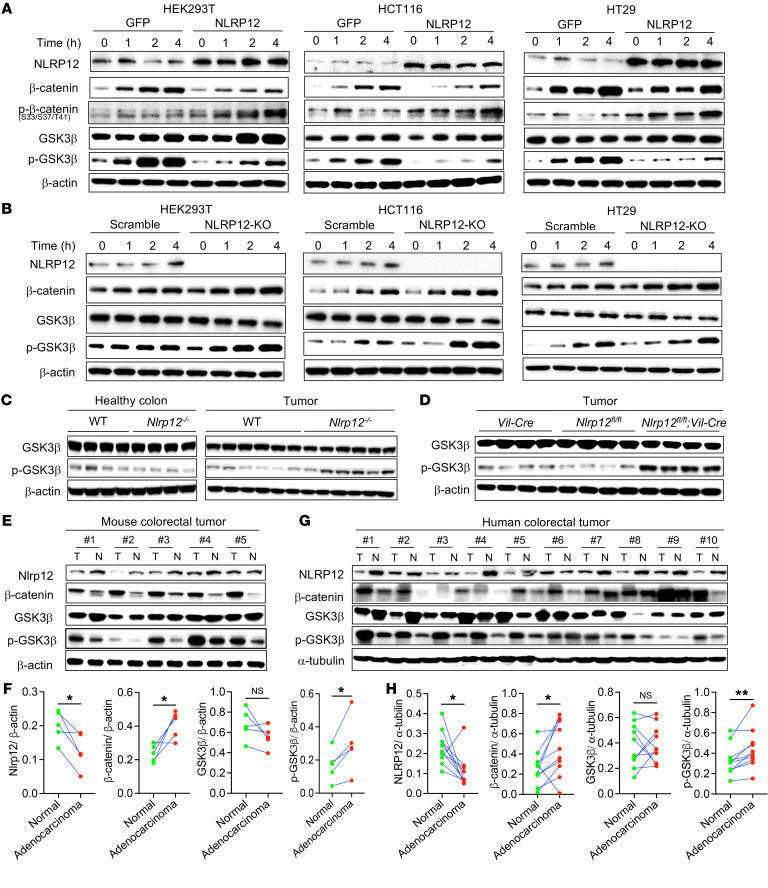
NLRP12 suppresses β-catenin activation via inhibition of GSK3β phosphorylation. (**A**) GFP- or NLRP12-expressing HEK293T, HCT116, and HT29 cells were stimulated with Wnt3a. Cell lysates were analyzed for NLRP12, β-catenin, p-β-catenin, GSK3β, p-GSK3β, and β-actin by Western blotting. (**B**) Scramble or *NLRP12*-KO HEK293T, HCT116, and HT29 cells were stimulated with Wnt3a. At indicated time points, cells lysates were used to measure NLRP12, β-catenin, GSK3β, p-GSK3β, and β-actin by Western blotting. (**C**) Colon tissues were collected from healthy and tumor-bearing WT and *Nlrp12^–/–^* mice and analyzed for GSK3β and p-GSK3β by Western blotting. (**D**) Colorectal tumors were induced in *Vil*-Cre, *Nlrp12^fl/fl^*, and *Nlrp12^fl/fl^*;*Vil*-Cre mice with AOM plus DSS. Tumors collected 12 weeks after AOM/DSS treatment were analyzed for GSK3β, p-GSK3β, and β-actin. (**E** and **F**) Colorectal tumors and adjacent nontumor tissues from WT mice following AOM/DSS-mediated tumor induction were used to measure NLRP12, β-catenin, GSK3β, p-GSK3β, and β-actin by Western blotting. (**F**) Band intensities of NLRP12, β-catenin, GSK3β, and p-GSK3β as shown in **E** were measured by densitometry. Data represent mean ± SEM. (**G** and **H**) Human colorectal tumors and adjacent nontumor tissues were analyzed for NLRP12, β-catenin, GSK3β, p-GSK3β and α-tubulin by Western blotting. Band intensities of NLRP12, β-catenin, GSK3β, and p-GSK3β were measured by densitometry. Data represent mean ± SEM. *P < 0.05, **P < 0.01 by unpaired, 2-tailed Student’s *t* test. Experiments represented in **A** and **B** were repeated 3 times, and those in **C**–**H** were repeated 2 times.

Next, we sought to explore the molecular processes involved in NLRP12-mediated suppression of β-catenin activation. In resting conditions, β-catenin is captured by the destruction complex and phosphorylated by GSK3β at the N-terminal Thr41, Ser37, and Ser33 residues (8). Phosphorylated β-catenin is ubiquitinated by β-TrCP ubiquitin E3 ligase, leading to its proteasomal degradation in the cytosol and inhibition of nuclear translocation of β-catenin (27, 29). Our data suggest that NLRP12 may facilitate ubiquitination-mediated degradation of β-catenin. Indeed, inhibition of ubiquitination of β-catenin by proteasome inhibitor MG132 increased the levels of total β-catenin and NLRP12-mediated degradation of β-catenin was rescued (Supplemental Figure 9A). Since ubiquitination of β-catenin is preceded by its phosphorylation by GSK3β, and GSK3β is inactivated by its phosphorylation during Wnt3a stimulation (6, 27), we measured the phospho-β-catenin (p-β-catenin) and p-GSK3β levels in HEK293T cells overexpressing NLRP12. Consistent with reduced levels of β-catenin, there was increased p-β-catenin at Ser33, Ser37, Thr41, and reduced p-GSK3β in NLRP12-overexpressing HEK293T cells (Figure 7A). Similarly, we observed reduced β-catenin and p-GSK3β in HCT116 and HT29 human CRC cells overexpressing NLRP12 (Figure 7A). Reduced phosphorylation of GSK3β was accompanied by its higher kinase activity in NLRP12-overexpressing HEK293T and HCT116 cells (Supplemental Figure 9B). These data suggest that NLRP12 inhibits phosphorylation of GSK3β and promotes phosphorylation and degradation of β-catenin.

To confirm the role of NLRP12 in suppressing the phosphorylation of GSK3β, we generated *NLRP12*-KO HEK293T, HCT116, and HT29 cells with CRISPR/Cas9 (Figure 7B). *NLRP12*-KO HEK293T cells showed higher levels of β-catenin as well as higher p-GSK3β following stimulation with Wnt3a (Figure 7B). Similarly, there was an increased level of β-catenin accompanied by increased p-GSK3β in Wnt3a-stimulated NLRP12-deficient HCT116, HT29, and MC38 cells (Figure 7B and Supplemental Figure 9C). To determine whether NLRP12 regulates GSK3β phosphorylation in primary cells, we grew organoids from WT and *Nlrp12^–/–^* small intestinal crypts and stimulated them with Wnt3a. *Nlrp12^–/–^* organoids exhibited higher phosphorylation of GSK3β (Supplemental Figure 9D). There was increased phosphorylation of GSK3β in *Nlrp12^–/–^* MEFs as well when stimulated with Wnt3a (Supplemental Figure 9E), further confirming the critical function of NLRP12 in regulating kinase activity of GSK3β.

### The expression of NLRP12 is inversely related to β-catenin activation and GSK3β phosphorylation in mouse and human CRC.

To examine in vivo evidence of NLRP12-mediated regulation of GSK3β phosphorylation, we measured p-GSK3β in tumors collected from AOM/DSS-treated WT and *Nlrp12^–/–^* mice (Figure 7C). As expected, there was increased p-GSK3β in *Nlrp12^–/–^* tumors, while no difference was seen in healthy WT and *Nlrp12^–/–^* colons (Figure 7C). Tumors from *Nlrp12^fl/fl^*;*Vil*-Cre mice also exhibited higher GSK3β phosphorylation as compared with those of control mice (Figure 7D), confirming the epithelial cell–specific role of NLRP12 in regulating the Wnt/β-catenin pathway. These data implied that dysfunction of NLRP12 in colorectal tumors facilitates β-catenin activation, promoting CRC. The link between NLRP12 and colorectal tumorigenesis was further evidenced by its expression profile in tumor and no-tumor tissue. Interestingly, the expression of NLRP12 was markedly reduced in colorectal tumors compared with adjacent nontumor tissues of WT mice (Figure 7, E and F, and Supplemental Figure 10, A and B). Consistent with reduced expression of NLRP12 in WT tumor, there was increased activation of β-catenin accompanied by higher phosphorylation of GSK3β in tumor tissue compared with adjacent nontumor tissue of WT mice (Figure 7, E and F). To confirm that NLRP12 expression is reduced explicitly in tumor epithelium, we isolated Ep-CAM^+^ cells from tumor and nontumor colon tissues of WT mice and measured NLRP12 at protein and RNA levels. Our data show reduced NLRP12 expression in Ep-CAM^+^ epithelial cells of tumor tissue compared with those of nontumor tissue (Supplemental Figure 10, C–E).

To understand the relevance of the observed connection between NLRP12 and β-catenin in human CRC, we measured the expression of NLRP12, β-catenin, and p-GSK3β in human colorectal adenocarcinoma and adjacent nontumor tissue. As we compared the expression of NLRP12 in adenocarcinoma relative to adjacent nontumor tissue, NLRP12 expression was significantly reduced in most cancer samples (9/10) compared with that of adjacent nontumor colon tissue (Figure 7, G and H, and Supplemental Figure 10F). Adenocarcinoma tissue with a reduced level of NLRP12 compared with adjacent tissue exhibited significantly higher β-catenin and p-GSK3β (Figure 7, G and H). Consistent with reduced NLRP12 and higher β-catenin, the expression of Wnt target genes was upregulated in tumor tissue compared with nontumor tissue (Supplemental Figure 10F). These data suggest that human colorectal adenoma is associated with reduced expression of NLRP12, which is inversely related to GSK3β phosphorylation and β-catenin activation.

### NLRP12 activates GSK3β and promotes β-catenin degradation via interaction with STK38.

**Figure 8:**
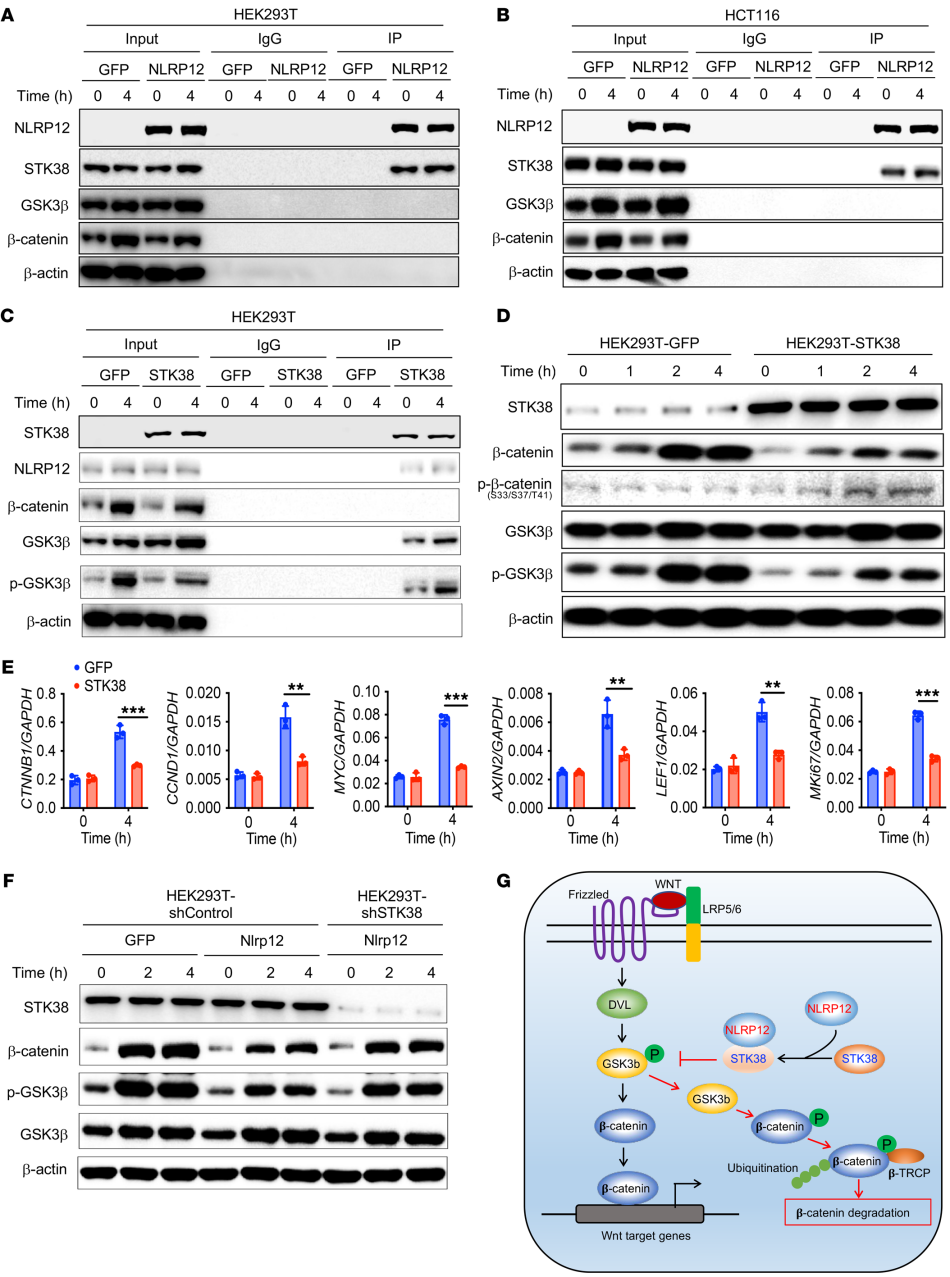
NLRP12 inhibits the phosphorylation of GSK3β via interaction with STK38. (**A** and **B**) HEK293T and HCT116 cells overexpressing FLAG-tagged NLRP12 or GFP were stimulated with Wnt3a. NLRP12 was pulled down with an anti-FLAG antibody, and the immunoprecipitation (IP) product and the input were immunoblotted for NLRP12, STK38, GSK3β, and β-catenin. (**C**–**E**) GFP-FLAG or STK38-FLAG was overexpressed in HEK293T cells and stimulated with Wnt3a. (**C**) Following IP of STK38, the IP product and the input were immunoblotted for STK38, GSK3β, p-GSK3β, NLRP12, and β-catenin. (**D**) Cell lysates were analyzed for STK38, β-catenin, p-β-catenin, GSK3β, and p-GSK3β. (**E**) The expression of indicated genes in HEK293T cells following stimulation with Wnt3a was measured by real-time RT-PCR. Data represent mean ± SD of triplicate wells. **P < 0.001, ***P < 0.0001 by unpaired, 2-tailed Student’s *t* test. (**F**) HEK293T cells expressing GFP or NLRP12 were transfected with shRNA for STK38 or control shRNA. Cells were then stimulated with Wnt3a and measured for STK38, β-catenin, p-GSK3β, and GSK3β by Western blotting. All experiments were repeated 3 times, and data of a representative experiment are presented. (**G**) Proposed mechanism of NLRP12-mediated inhibition of the Wnt/β-catenin pathway.

Given that GSK3β is an important kinase and plays a central role in the regulation of β-catenin, we were intrigued by how NLRP12 inhibits phosphorylation of GSK3β. To verify the possible interaction of NLRP12 with GSK3β, we overexpressed FLAG-tagged NLRP12 in HEK293T cells. After pulling down NLRP12, the immunoprecipitation product was analyzed by mass spectrometry (MS). Interestingly, we did not find GSK3β, β-catenin, or other signaling adapters of the Wnt/β-catenin pathway among the interacting partners of NLRP12 (data not shown). However, we identified STK38 and STK38-like protein (STK38L) among the proteins identified by MS. We confirmed the interaction of NLRP12 with STK38 by proteomic approaches following overexpression of NLRP12 and STK38 in HEK293T cells. NLRP12 was coimmunoprecipitated with STK38 as detected by Western blotting (Supplemental Figure 11A). STK38, also known as NDR1, a member of PKA/PKG/PKC-like (AGC) family, possesses both kinase and phosphatase activity (30–32). We, therefore, postulated that the interaction of NLRP12 with STK38 leads to dephosphorylation of GSK3β, and subsequent phosphorylation of β-catenin. To test this hypothesis, we overexpressed NLRP12 in HEK293T and HCT116 cells and stimulated them with Wnt3a. NLRP12 was immunoprecipitated and the product was immunoblotted for NLRP12, STK38, GSK3β, and β-catenin. Only STK38, but not GSK3β and β-catenin, interacted with NLRP12 (Figure 8, A and B). We repeated interaction studies following the overexpression of STK38 in HEK293T cells. Impressively, both NLRP12 and GSK3β but not β-catenin were coimmunoprecipitated with STK38 (Figure 8C). Notably, the interaction of STK38 with GSK3β was increased after Wnt3a stimulation (Figure 8C).

Next, we wanted to address whether STK38 is a regulator of the Wnt/β-catenin pathway. To this end, we overexpressed STK38 in HEK239T cells and measured phosphorylation of β-catenin and GS3Kβ phosphorylation following stimulation with Wnt3a. Consistent with the result observed in NLRP12-overexpressing cells (Figure 7A), STK38 overexpression resulted in increased phosphorylation and degradation of β-catenin (Figure 8D). These events were associated with decreased phosphorylation of GSK3β (Figure 8D), leading to increased kinase activity of GSK3β (Supplemental Figure 11B) in STK38-expressing cells. Reduced expression of Wnt targets genes, including *CTNNB1*, *CCND1*, *MYC*, *AXIN2*, *LEF1*, and *MKi67*, in HEK293T-STK38 cells further confirmed that STK38, like NLRP12, suppresses the Wnt/β-catenin pathway (Figure 8E).

Finally, we examined whether NLRP12-mediated regulation of Wnt/β-catenin is STK38 dependent or not. Hence, we knocked down STK38 in NLRP12-overexpressing HEK293T cells. Control or STK38-knockdown cells were then stimulated with Wnt3a and analyzed for the activation of β-catenin. As expected, β-catenin activation and GSK3β phosphorylation were reduced in NLRP12-overexpressing cells (Figure 8F). However, such downregulation of β-catenin activation and GSK3β phosphorylation were rescued when STK38 expression was lowered with shRNA (Figure 8F), supporting the notion that NLRP12-mediated inhibition of GSK3β phosphorylation and subsequent degradation of β-catenin are mediated through STK38. Notably, the expression of STK38 was not affected by the absence of NLRP12 in the tumor epithelium (Supplemental Figure 11, C–E), suggesting that NLRP12 regulates the Wnt/β-catenin pathway through its interaction with STK38 but not the regulation of STK38 expression. Taken together, these data revealed a regulatory mechanism of the Wnt/β-catenin pathway in which NLRP12 inhibits phosphorylation of GSK3β via interacting with STK38, leading to phosphorylation and proteasomal degradation of β-catenin (Figure 8G).

## Discussion

The Wnt/β-catenin pathway plays many essential functions in the intestine, including proliferation and differentiation of intestinal epithelial cells, maintenance of tight junctions, and cell-to-cell communication (4, 5, 33). However, excessive activation of this pathway causes neoplastic transformation of epithelial cells, as well as promotion of tumor growth, angiogenesis, tumor invasion, and metastasis (9). While mutations in *APC* or other components of the β-catenin destruction complex are primarily responsible for hyperactivated β-catenin, several other molecules, such as DKK1, WIF1, Sox17, etc., regulate β-catenin activation and are linked to CRC pathogenesis (34–37). Here, we describe a regulatory mechanism of the Wnt/β-catenin pathway in which NLRP12 promotes the degradation of β-catenin.

The involvement of NLRP12 in various disease pathogenesis is increasingly evident. *Nlrp12^–/–^* mice are susceptible to colitis, colorectal tumorigenesis, autoimmune encephalomyelitis, and hepatocellular carcinoma, and exhibit protection against bacterial infection and hemolytic disease (13–17, 19, 38). NLRP12-mediated protection against inflammatory disorders was attributed to its role in attenuating inflammatory responses downstream of NF-κB and MAPK pathways (13, 14, 16, 17). However, recent studies suggest that NLRP12’s role in diverse pathophysiology is beyond its regulation of NF-κB and MAPK pathways. For example, NLRP12 is involved in the inflammasome and PANoptosome formation, activation of RIGI, and ubiquitination of NOD2 (19, 39–41). The findings of this study support this notion by adding a previously unknown function of NLRP12.

Given the known function of NLRP12 in suppressing NF-κB and MAPK pathways (13, 16, 17), it is surprising that these inflammatory pathways were not hyperactivated in *Nlrp12^–/–^* tumors. Previous studies showed higher activation of NF-κB, ERK, and STAT3 in the colon of *Nlrp12^–/–^* mice during acute DSS-induced colitis (13, 17). Inflammation is a well-known trigger for tumor growth; thus, higher inflammation and activation of inflammatory signaling pathways were considered the underlying cause of increased tumorigenesis in *Nlrp12^–/–^* mice (13, 17). However, the data of this study imply that NLRP12 downregulates NF-κB and MAPK pathways in the colon during acute colitis, but not chronic colitis or tumorigenesis. Notably, although NF-κB and MAPK pathways were not dysregulated, there was increased STAT3 activation in colorectal tumors of *Nlrp12^–/–^* mice, which could be secondary to increased β-catenin (42). However, it remains unclear why NF-κB and MAPK signaling pathways are not hyperactivated in *Nlrp12^–/–^* tumors. It is possible that other negative regulators of NF-κB and MAPK are induced in the inflamed colon in the absence of NLRP12 to mitigate the hyperinflammatory responses therein.

Our study with *Nlrp12*–conditional KO mice suggests that NLRP12-mediated regulation of the Wnt/β-catenin pathway in intestinal epithelial cells is critical in suppressing colorectal tumorigenesis. However, this observation does not rule out the immune cell–specific function of NLRP12 in the protection against colorectal tumorigenesis. Hyperinflammatory responses at the early stages of tumorigenesis may also lead to a higher tumor burden in *Nlrp12^–/–^* mice. Indeed, in our earlier study, we observed an increased tumor burden in bone marrow chimera mice having NLRP12 deficiency in the hematopoietic compartment (17). Since NLRP12 downregulates inflammatory responses in myeloid cells and lymphocytes (13, 14, 16, 17, 43), it would be interesting to determine the role of NLRP12 in different immune cells in the regulation of colorectal tumorigenesis using respective conditional KO mice.

While our findings demonstrate the intestinal epithelial cell–specific antitumor function of NLRP12, it is intriguing why such a role was not observed in a previous bone marrow chimera study (17). This apparent discrepancy underscores the limitations of using bone marrow chimeras to study the cell-specific function of a gene. Bone marrow chimera generation involves whole-body irradiation with ionizing radiation, which causes damage to the gastrointestinal tract, death of intestinal stem cells, malabsorption, and changes in the gut microbiota composition in mice (44, 45). Radiation-exposed epithelial cells also express genes differentially, affecting cellular proliferation and stress responses (44). Therefore, the intestinal epithelium of irradiated mice may develop resistance to tumorigenesis. Indeed, bone marrow chimeric mice developed a very low number of colorectal tumors with AOM/DSS treatment (17). Due to the molecular and biochemical changes in the intestinal epithelial cells of irradiated mice, it is likely that the epithelial cell–intrinsic antitumor function of NLRP12 was not evident in the chimeric mice.

NLRP12 in different diseases is mostly implicated in immune cell–specific antiinflammatory responses (13, 14, 17, 18, 39, 46). Here, we demonstrate a nonimmune function of NLRP12 involved in the regulation of GSK3β, which is a major kinase regulating hundreds of signaling adapters (47). Our data suggest that NLRP12 does not directly activate GSK3β, but does so through its interaction with STK38. However, the precise mechanism through which STK38 regulates phosphorylation of GSK3β is yet to be explored. Previous studies showed that STK38 interacts with MEKK1/2, upstream activators of MAPK pathways, leading to the inhibition of its catalytic function (48, 49). STK38-mediated inhibition of MEKK1/2 involves inhibition of autophosphorylation and promotion of dephosphorylation of MEKK1/2 (48). Furthermore, STK38 promotes the degradation of MEKK1/2 via interaction with E3 ubiquitin ligase Smurf1 (49). STK38 may inactivate GSK3β through a similar mechanism. The interaction of STK38 with GSK3β was shown by an earlier study suggesting that the kinase activity of STK38 is inhibited by GSK3β during oxidative stress–induced apoptosis (50). It is possible that the interaction of STK38 with GSK3β leads to the inhibition of kinase activity of either GSK3β or STK38, depending on the physiological contexts or interaction site. While GSK3β is phosphorylated by several other kinases, it can be autophosphorylated as well (51–54). Reduced p-GSK3β in STK38-overexpressing cells, therefore, suggests that STK38 inhibits either autophosphorylation of GSK3β or its phosphorylation by other kinases. The precise mechanism through which STK38 regulates phosphorylation of GSK3β and β-catenin stability should be investigated in the future.

STK38/NDR1 has recently emerged as a critical regulator of the cell cycle, development, apoptosis, and cancer (48, 55–58). The diverse biological functions of STK38 are dependent on its activation, which is mediated by autophosphorylation at Ser281 and phosphorylation at Thr444 by other kinases (31, 59). At homeostasis, STK38 activity remains low due to its dephosphorylation by protein phosphatase 2A (59). It has been shown that STK38 interacts with several proteins, including S100B, hMOB1, SOCS2, MEKK1/2, and GSK3β, while exerting its biological functions (31, 60–63). Our findings suggest that NLRP12 is another potential signaling partner of STK38. It is possible that NLRP12 inhibits dephosphorylation of STK38 or facilitates its phosphorylation. Future studies should dissect the mechanism of how NLRP12 regulates the kinase activity of STK38.

In summary, this study mechanistically demonstrates that NLRP12 in the tumor epithelium plays a pivotal role in regulating their proliferation, invasion, and migration. Although NLRP12 is not a classical cancer suppressor, our data suggest that genetic alteration or reduced expression of NLRP12 may facilitate tumorigenesis and cancer invasiveness. This study also elucidates what we believe is a new regulatory mechanism of the Wnt/β-catenin pathway involving NLRP12 and STK38. The observed connection between NLRP12 expression and inhibition of β-catenin activation opens opportunities for developing new therapeutic interventions for CRC targeting the NLRP12/STK38/GSK3β signaling axis.

## Methods

Additional detail can be found in Supplemental Methods.

### Mice.

C57BL/6J (WT) and *APC^min/+^* mice were purchased from The Jackson Laboratory. *Nlrp12^–/–^* mice were generated by Millennium Pharmaceuticals. *Nlrp12^fl/fl^* mice were generated by our laboratory with the help of the UT Southwestern Mouse Transgenic Core facility. The background of all mice used in this study is C57BL/6J. Unless otherwise stated, mice of different genetic backgrounds were housed in separate cages, maintained in the same animal room, and used for in vivo and in vitro experiments. All mice were bred and maintained in a specific pathogen–free (SPF) facility at the UT Southwestern Medical Center. For cohousing experiments, 4-week-old WT mice were cohoused with *Nlrp12^–/–^* mice for 30 days. All animal studies were conducted with age- and sex-matched mice.

### Induction of colorectal tumorigenesis.

Colorectal tumorigenesis was induced in mice using the AOM plus DSS model, as described previously (17). Briefly, mice were injected AOM (Sigma-Aldrich) intraperitoneally (10 mg/kg body weight) and maintained on regular drinking water. Five days after AOM injection, mice were administered ad libitum with 2.5% DSS (molecular mass, 36–40 kDa; TdB Consultancy) for 5 days, followed by regular drinking water for 10 days. The DSS cycle was repeated 2 more times. Mice were sacrificed 10 to 12 weeks after AOM injection.

### Real-time RT-PCR.

Tumors, colons, and small intestines from mice were preserved in RNAlater (Invitrogen). Cultured cells and tissues were lysed in TRIzol reagent (Invitrogen). Total RNA was extracted using TRIzol reagent (Invitrogen) according to the manufacturer’s instructions. Isolated RNA was reverse transcribed into cDNA using iScript (Bio-Rad). Real-time RT-PCR was performed using iTaq Universal SYBR Green Supermix (Bio-Rad). Expression data were normalized to *Gapdh* as described previously (64). Primers used for real-time RT-PCR are listed in Supplemental Table 1.

### Western blot analyses.

Mouse and human tissues or cultured cells were homogenized in RIPA lysis buffer containing complete protease inhibitor and phosphatase inhibitor cocktail (Roche), resolved by SDS-PAGE, and transferred onto a PVDF membrane. The membranes were immunoblotted with antibodies against p-IκBα (9246, Cell Signaling Technology [CST]), IκBα (4812, CST), NLRP12 (OAAB04256, Aviva Systems Biology), p-ERK (4370, CST), ERK (4695, CST), p-JNK (4668, CST), p-AKT (4060, CST), β-catenin (8480, CST), p-β-catenin (9561, CST), GSK-3β (9315, CST), p-GSK-3β Ser9 (9323, CST), STK38 (H00011329-M01, Abnova), p-STAT3 (9145, CST), p-NF-κB p65 (3033, CST), cMyc (5605, CST), lamin B1 (13435, CST), cyclin D1 (2978, CST), FLAG M2 (F1804, Sigma-Aldrich), α-tubulin (3873, CST), Myc (2376, CST), and β-actin (A2228, Sigma-Aldrich). Immunoreactive proteins were detected using ECL Super Signal West Femto substrate reagent (Thermo Fisher Scientific).

### Statistics.

Data are presented as mean ± SD or ± SEM (as stated in the figure legends). All animal studies were repeated at least 2 times and in vitro studies were repeated 3 times. Statistical significance when comparing 2 groups was determined by 2-tailed, unpaired Student’s *t* test. One-way ANOVA was used to analyze statistical significances between multiple groups. A *P* value of less than 0.05 was considered statistically significant. GraphPad Prism 9 software was used for statistical analyses.

### Study approval.

All animal experiments were approved by the Institutional Animal Care and Use Committee (IACUC) of UT Southwestern Medical Center and were conducted in according with the guidelines of IACUC and the NIH *Guide for the Care and Use of Laboratory Animals* (National Academies Press, 2011). All human tissue used in this study was provided by the Tissue Management and Shared Resource, an IRB-approved core facility of Simmons Comprehensive Cancer Center, UT Southwestern Medical Center.

### Data availability.

The 16S rRNA gene sequence data have been deposited to NCBI BioProject database under Bioproject PRJNA628078. The RNA-seq data have been deposited to NCBI Gene Expression Omnibus (GEO) and is publicly available with the accession number GSE149769. Values for all data points used in graphs can be found in the supplemental Supporting Data Values file.

## Author contributions

HZ conceived and designed the study, performed experiments, analyzed data, and wrote the manuscript. SK performed experiments, analyzed data, and wrote the initial draft of the manuscript. YTK and SH performed experiments. LP performed histopathological examination. BLC performed analysis of 16S rRNA gene sequencing data. YG and RSM helped in RNA-seq data analysis. CML provided human colorectal cancer samples. TDK provided *Nlrp12^–/–^* mice and reviewed the manuscript.

## Supplementary Material

Supplemental data

Supporting data values

## Version 1 08/15/2023

In-Press Preview

## Version 2 10/02/2023

Electronic publication
