# Supplementary material for: NLRP12 downregulates the Wnt/β-catenin pathway via interaction with STK38 to suppress colorectal cancer
Source: J Clin Invest. 2023 Oct 2;133(19):e166295. doi: 10.1172/JCI166295 (PMC10541192; doi:10.1172/JCI166295)
Supplement: Supplemental data [file jci-133-166295-s185.pdf]

Supplemental Materials

NLRP12 downregulates the Wnt/β-catenin pathway via interaction with STK38 to suppress colorectal cancer

Shahanshah Khan, Youn-Tae Kwak, Lan Peng, Shuiqing Hu, Brandi L. Cantarel, Cheryl M. Lewis, Yunpeng Gao, Ram S. Mani, Thirumala-Devi Kanneganti, and Hasan Zaki

Supplemental Methods

Generation of Nlrp12-conditional knockout mice. *Nlrp12*$^{flox/flox}$ mice were generated at UT Southwestern Transgenic Core facility by inserting loxP sites flanking 615 bp in exon 4 of *Nlrp12* using CRISPR/Cas9 system. Two sgRNAs were designed to separately target upstream and downstream of the target sequence. sgRNA was designed, validated, and synthesized by Sigma. The ss oligo donor containing loxP sequences and gRNA target sequence was synthesized by GENEWIZ. gRNA, oligo donors, and CRISPR reagents were mixed and injected into fertilized zygotes of C57BL/6J mice, and blastocysts were transferred into recipient female mice. The transgenic founders were screened by genotyping PCR. Founders with correct insertion of LoxP sites were identified by gene sequencing following PCR using primers targeting both LoxP sites. The founder was crossed with C57BL/6J mice and *Nlrp12*$^{flox/flox}$ mice were maintained in the animal facility at UT Southwestern. The *Nlrp12*$^{flox/flox}$ mice were then bred with *Vil-Cre* mice (Jackson, #004586) mice to generate intestinal epithelial cell-specific Nlrp12 knockout mice (*Nlrp12*$^{flox/flox}$*; Vil-Cre*).

Culture and stimulation of mouse embryonic fibroblasts. Mouse embryonic fibroblasts were isolated and cultured, as described previously (1) with minor modifications. Briefly, embryos were

harvested from 13–14 days old pregnant WT and *Nlrp12*$^{-/-}$ mice through cervical dislocation. After removing the embryos head, heart and liver, the remaining embryos were chopped in 0.25% trypsin-EDTA with sterile scissors and resuspended using a pipet. The cell suspension was poured in a Petri dish and incubated at 37°C for 10 min. The cell suspension was mixed well with pipetting several times and incubated for another 10 min at 37°C. Cells were transferred in a 50ml tube containing 20ml DMEM plus 10% FBS and 1% Pen/Strep (MEFs media). After several times of pipetting, the unsuspended tissues were allowed to settle down for 5 min. The supernatant containing single cells was transferred into a T75 flask, which was then incubated at 37°C with 5% CO_2. WT and *Nlrp12*$^{-/-}$ MEFs were seeded in a 12-well plate and stimulated with Wnt3a (100 ng/ml). After washing with chilled-PBS, MEFs were lysed with RIPA and Trizol for protein and RNA, respectively.

Fecal microbiota transplantation. Fecal homogenate from WT and *Nlrp12*$^{-/-}$ mice were orally gavaged into germ-free (GF) mice as described previously (2). Briefly, fresh stool from WT and *Nlrp12*$^{-/-}$ mice were collected and homogenized in 3% thioglycolate broth (20% W/V). Stool homogenates were centrifuged at 1000 rpm, and supernatants were orally gavaged into GF mice every alternate day for a total of 3 doses. Two weeks following the final fecal administration, mice were subjected to AOM-DSS treatment.

Histopathology and immunohistochemistry. Following dissection, colons were washed with pre-chilled PBS, fixed in 4% paraformaldehyde, embedded in paraffin, and stained with H&E. Histological scoring analysis was performed in a blinded fashion by a pathologist. Tumors were graded as low-grade dysplasia, high-grade dysplasia, and invasive adenocarcinoma. Scoring criteria for tumor histopathology was described previously (3). For immunohistochemistry, 4%-paraformaldehyde-fixed and paraffin-embedded colon tissue sections were deparaffinized and hydrated by sequential dipping into 100%, 95%, 90%, 80% and 70% ethanol. Heat-induced antigen retrieval was performed in 10mM

sodium citrate solution (pH 6.0) for 20 min at 95°C. Tissue sections were blocked with 5% goat serum prepared in PBS plus 0.05% Tween 20 (PBST) for 30 min at room temperature and incubated with anti-β-catenin (8480; Cell Signaling) and anti-Ki67 (ab16667; Abcam) antibodies overnight at 4°C. The tissue sections were then washed three times in PBST and incubated with secondary antibody Cy3-conjugated goat anti-rabbit IgG (Life technologies, A10520) or HRP-conjugate anti-rabbit (Sigma Aldrich, A0545) for 2h in the dark. Following 3 washes, tissue sections were mounted with mounting media with or without DAPI.

Human colon adenocarcinoma tissues. Ten pairs of human colon adenocarcinoma and adjacent normal mucosa snap-frozen tissues were collected from UT Southwestern Medical Center (UTSW) Tissue Management Shared Resource on approval of the Institutional Review Committee Board. These tissue samples were belongings to high-grade adenocarcinoma patients who underwent surgical removal of colorectal cancer at UTSW. The age of these patients ranged between 44 to 80 years (average age 62.8), while tumor size ranged between 3.2 cm to 13.5 cm (average size 6.32 cm). The details of these high-grade adenocarcinoma patients with clinical data are listed in Table S3.

16S rRNA sequencing. Using the Fecal DNA isolation kit (Qiagen, USA), total genomic DNA was extracted from fecal pellets. The quality of DNA was assessed by agarose gel electrophoresis. Bacterial primers 341F 5'-CCTACGGGAGGCAGCAG-3') and 806R (5'-GGACTACHVGGGTWTCTAAT-3') targeting the V3-V4 hyper-variable region of 16S rRNA gene were used for PCR amplification. In library preparation, sample-specific barcode sequences were incorporated to the primers, and then barcoded 16S rRNA gene amplicons were pooled at equimolar concentration prior to sequencing using MiSeq platform (Illumina, Inc., San Diego, California). 16S sequencing was performed by UT Southwestern Microbiome Genomics Core facility. The 16S rRNA gene sequence raw data was analyzed by MOTHUR protocol (4) with the following modifications: (i) sequences were filtered using max length optimizations of 90%, and

(ii) alpha diversity was calculated using Inverse Simpson and Shannon Diversity. The 16S rRNA gene sequence data have been deposited to NCBI BioProject database under Bioproject PRJNA628078.

RNA-seq analysis. RNA sequencing was performed by Novogene (CA). Quality control of RNA-seq data was performed by removing reads containing adapters, low quality (Qscore<= 5) and N > 10% (N represents the base cannot be determined). Sequencing Reads were mapped to hg19 using STAR (5), and gene expression levels for each sample were calculated by RSEM (6) with default setting. Differential Expression Analysis was conducted by EBSeq (7) with FDR=0.05. Heatmap was plotted using normalized expression outputs from RSEM. The raw RNA-seq data have been deposited to NCBI Gene Expression Omnibus (GEO) and is publicly available with the accession number GSE149769.

Cell culture. The human embryonic kidney epithelial cell line HEK293T (ATCC, CRL-3216), and murine colon adenocarcinoma cell line MC38 (shared by Dr. Yang-Xin Fu, UT Southwestern Medical Center) were cultured in Dulbecco's Modified Eagle's medium (DMEM; Sigma, Cat. No. D6429) supplemented with 10% (v/v) FBS (Sigma, Cat. No. F4135) and 1% (v/v) Pen/Strep (Gibco, Cat. No. 15070063) and maintained in a 5% CO_2 incubator at 37°C. Human colorectal carcinoma cells HCT116 (ATCC, CCL-247) and HT-29 (ATCC, HTB-38) were cultured in McCoy`s 5A medium (Gibco, Cat. No. 16600082) supplemented with 10% (v/v) FBS (Sigma, Cat. No. D6429) and 1% (v/v) Pen/Strep (Gibco, Cat. No. 15070063) and maintained in a 5% CO_2 incubator at 37°C. Each cell line was confirmed free from mycoplasma contamination by testing with mycoplasma detection kit (InvivoGen, rep-mys-50).

Isolation and 3D culture of intestinal crypts. Mouse intestinal crypts were cultured in vitro to form 3D organoids, as described previously (8). Briefly, small intestines (SI) were collected,

washed vigorously in ice-cold PBS, cut longitudinally and incubated in 1mM DTT for 10 min to remove mucin layer. SI were then cut into small pieces and incubated with 5 mM EDTA in HBSS buffer containing 100 u/ml penicillin/streptomycin and 20 µg/ml gentamicin with gentle shaking. For isolation of SI crypts, the villus fraction was removed after 10 min of incubation. Remaining crypt fractions were incubated with fresh 5 mM EDTA buffer for an additional 15 min at 37°C. Isolated epithelial cells and crypts were passed through 70 µm cell-strainer and washed several times in ice-cold HBSS by centrifugation at 500 rpm. The isolated crypts (2000 crypts/well of 24-well) were mixed with matrigel and placed on the center of each 24-well cell culture plate. The plate was incubated for 10 min at 37°C to solidify the matrigel. A total of 500µl basal medium (advanced DMEM/F12 medium, 10mM hepes, 2mM glutamax, 100µg/ml primocin) supplemented with N2 (1X, Invitrogen), B27 (1X, Invitrogen), Noggin (100ng/ml, Peprotech), EGF (50ng/ml, Peprotech), R-spondin (1ug/ml, Peprotech), N-acetyl cysteine (50ng/ml, Sigma), penicillin-streptomycin (100u/ml) and Y-27623 (10mM/ml) were added to each 24-well culture plate. The fresh medium was added to each well every 48h. The number of well-formed organoids per well was counted. The size of the crypts was measured by analyzing the images captured under 40X microscopic field in a Zeiss microscope. For stimulation, organoides were washed and incubated for 4h in a basal medium (without any growth factors and Rspondin). Cells were then stimulated with Wnt3a (100ng/ml).

cDNA constructs and transient transfection. The Flag sequence was cloned in pcDNA4/TO vector at XhoI and ApaI sites. Then, the full-length mouse Nlrp12 cDNA was cloned at the KpnI and NotI site into the pcDNA4/TO:Flag vector. Full length of human NLRP12 cDNA was cloned into the flag-tagged pcDNA4/TO vector at the sites of HindIII and EcoRV. Full length of human NLRP3 cDNA was cloned at BamHI and XhoI into the flag-tagged pcDNA4/TO vector. Human NLRP6 was cloned at the site of EcoRI and XhoI into the flag-tagged pcDNA4/TO vector. Full

length of human STK38 cDNA was cloned either at HindIII and PstI or HindII and BamHI sites into the Flag-tagged or Myc-tagged pcDNA4/TO vector, respectively. As a control, GFP was cloned into the pcDNA4/TO:Flag vector at BamHI and NotI sites. At 50 – 60 % confluency, HEK293T, HT-29 and HCT116 cells were transfected with NLRP12-Flag or GFP-Flag (1 μg/ml) constructs using Lipofectamine 3000 reagent (Invitrogen) according to manufacturer`s instructions, and confirmed by observing GFP under fluorescence microscope and Western blot analysis of Nlrp12. After 48h post-transfection, cells were stimulated with Wnt3a (100ng/ml). Cells were lysed with RIPA lysis buffer containing complete protease inhibitor cocktail and phosphatase inhibitor cocktail (Roche) for protein (Western blot) or with TRIzol™ reagent (Invitrogen) for RNA (real-time RT-PCR).

Overexpression and immunoprecipitation of NLRP12 for mass spectrometry analysis. A CMV expression vector encoding Flag-tagged NLRP12 was transfected into HEK293T cells to establish a stable cell line. Affinity-purified Flag-tagged NLRP12 protein and associated proteins were isolated from HEK293T cells stably expressing Flag-tagged NLRP12. The cells were harvested and homogenized in Tris-buffered saline (TBS) (50 mM Tris HCl [pH 7.4], 150 mM NaCl). After centrifugation at 3,000 rpm for 10 min in a CS-6R Beckman centrifuge, the supernatant was applied to an anti-Flag M2 affinity gel column (Sigma) as specified by the manufacturer. Flag-tagged NLRP12 and associated proteins were eluted with TBS containing Flag peptide (Sigma) at 100 μg/ml. The eluted proteins were digested with trypsin and precipitated with TCA, and the peptides were analyzed by matrix-assisted laser description ionization time-of-flight (MALDI-TOF) mass spectra.

Cytosolic and nuclear subcellular fractionation. Nuclear and cytoplasmic protein extracts were separated by NE-PER nuclear and cytoplasmic extraction reagents (Thermo Fisher

Scientific, 78835) according to manufacturer instructions. Briefly, the cell pellet was suspended in ice-cold CER I reagent supplemented with protease inhibitor cocktail and phosphatase inhibitor cocktails (Roche). After incubation on ice for 10 minutes, the CER II reagent was added, and the cells were lysed by vortexing. The homogenate was centrifuged for 5 minutes at 16,000 x g. The supernatant representing the cytosolic fraction was collected, and the pellet containing the cellular nuclei was dissolved in ice-cold NER reagent supplemented with protease and phosphatase inhibitors. The Eppendorf tube containing the nuclear fraction was incubated for 40 min on ice and vortexed for 15 seconds in every 10 minutes. After centrifugation for 10 min at 16,000 x g at 4°C, the supernatant (nuclear fraction) was transferred to a clean pre-chilled tube. The cytoplasmic and nuclear fractions were resolved by SDS-PAGE.

STK38 knockdown in HEK293T cells using siRNA. HEK293T cells at 70% confluency were transfected with MISSION® pLKO.1-puro (shControl, Sigma) or STK38 specific SiRNA (Clone ID: NM_007271.21231s21c1, Sigma) using Lipofectamine 3000 reagent (Invitrogen) according to manufacturer`s instructions. 48h post transfection, co-transfected HEK293T cells were selected using media containing 2 μg/ml puromycin (A1113803, Gibco). The STK38 knockdown in HEK293T cells was further confirmed by Western Blot analysis. The selected STK38 knockdown-HEK293T and shControl-HEK293T cells were seeded in a 12-well plate for overnight and stimulated with Wnt3a (100ng/ml). After 1X washing with pre-chilled PBS, cells were lysed with RIPA lysis buffer containing complete protease inhibitor and phosphatase inhibitor cocktails (Roche).

Immunofluorescent staining. HEK293T cells were grown on coverslips and transfected with GFP and NLRP12 plasmids using Lipofectamine 3000 reagent (Invitrogen) according to manufacturer`s instructions. 48h post transfection, cells were stimulated with Wnt3a (100ng/ml) for 2h, and fixed in 4% paraformaldehyde for 15 min at RT, washed 3X with PBS for 5 min each

and blocked with PBS containing 5% goat normal serum and 0.3% Triton-X100 for 1h. Cells were then incubated with primary antibodies against β-catenin (8480, Cell Signaling) overnight at 4°C. After 3X washing in PBS, cells were incubated with secondary antibody Cy3-conjugated goat anti-rabbit IgG (Life technologies, A10520) for 1h at RT. Following three washes in PBS, cells were mounted with mounting media containing DAPI. Images were taken with a fluorescence microscope (Zeiss).

***Co-immunoprecipitation*.** HEK293T and HCT116 cells were transfected with Flag-tagged GFP, NLRP12, or STK38 (1μg/ml) plasmids using Lipofectamine 3000 reagent (Invitrogen) according to manufacturer`s instructions. 48h post-transfection, cells were stimulated with Wnt3a (100ng/ml) for various times and lysed in Peirce® IP lysis buffer supplemented with complete protease inhibitor cocktail and phosphatase inhibitor cocktail (Roche), according to manufacturer`s instruction (87787, ThermoFisher). Cell lysates were centrifuged to pellet cell debris, and the supernatants were processed for immunoprecipitation using anti-Flag®M2 (F1804, Sigma-Aldrich) or anti-Myc (2376, Cell Signaling) antibodies with Dynabeads® Protein G according to manufacturer`s protocol (Novex® by Life Technologies™).

Generation of Nlrp12 knockout cells using CRISPR/Cas9. At 50 – 60 % confluency, HEK293T, HCT116 or HT-29 cells were transfected with scrambled sgRNA CRISPR/Cas9 (abm-K010), NLRP12 sgRNA CRISPR/Cas9 (abm-K1434706 and abm-K1434708, accession number: NM_144687) plasmids using Lipofectamine 3000 reagent (invitrogen) according to manufacturer`s instructions. MC38 cells were transfected with scrambled sgRNA CRISPR/Cas9 (VB180911-1190rqd) and Nlrp12 two-sgRNA CRISPR/Case9 (VB190527-1100acq, accession number: NM_001033431.1) plasmids using Lipofectamine 3000 reagent (invitrogen) according to manufacturer`s instructions. After 48h post-transfection, CRISPR/Cas9 plasmids co-transfected HEK293T, HCT116, HT-29, and MC38 cells were selected using media containing 2 μg/ml

puromycin (A1113803, Gibco). The NLRP12 knockout HEK293T, HCT116, HT-29, and MC38 cells were further confirmed by Western blot analysis. The selected NLRP12 knockout HEK293T, HCT116, HT-29, or MC38 and scrambled HEK293T, HCT116, HT-29, or MC38 cells were seeded in 12-well plates for overnight and stimulated with Wnt3a (100ng/ml), as indicated time points. After washing with pre-chilled PBS once, cells were lysed with RIPA lysis buffer containing complete protease inhibitor and phosphatase inhibitor cocktails (Roche).

Real-time cell proliferation by IncuCyte live-cell imaging system. Scrambled MC38 and Nlrp12 knockout MC38 cells were seeded in 96-well plate ($1.5x10^3$/ well) and incubated at 37°C in a humidified CO_2 incubator. 6h following seeding, the plate was placed into the IncuCyte live-cell imaging system under 10x objective with four fields imaged per well. The real-time cell proliferation was measured at 6h intervals (9). The percentage of cell confluence was automatically calculated by analyzing the images captured by the IncuCyte software (Essen Bioscience, Ann Arbor, MI, USA).

Migration and invasion assay using IncuCyte live-cell imaging system. About $2x10^4$ MC38 (Scramble) and Nlrp12 knockout (Nlrp12-KO) MC38 cells were seeded in 96-well ImageLock microplate (Essen Bioscience) and allowed overnight to form a uniform monolayer. The uniform and reproducible scratches were created using the WoundMaker (Essen Bioscience) in the 96-well ImageLock microplate. The medium is aspirated, and the wells were washed two times with fresh medium to remove any detached cells from the scratched area. Following the final wash, 100 μL of fresh medium was added to each well of the ImageLock plate containing cells and then placed into the IncuCyte live-cell imaging system. The real-time scratch closure images were captured every 2h intervals by IncuCyte software (Essen Bioscience). The percentage of wound density and percentage of wound confluence were automatically calculated and analyzed by the

IncuCyte software. The percent wound density represents the cell density in the scratched area relative to the outside cell density over time. Similarly, percent wound confluence is the cell confluence in the scratched area over time.

Clonogenic assay. Scrambled and Nlrp12 knockout MC38 cells were seeded at a density of $2x10^2$ cells/well of a 6-well plate. At day 9, culture medium was aspirated and cells were washed with PBS. The cells were then fixed and stained with a mixture of 6% glutaraldehyde and 0.5% crystal violet for 30 min at room temperature. The glutaraldehyde crystal violet mixture was washed with tap water and the plate was air-dried.

GSK3β kinase assay. HEK293T and HCT116 cells were stimulated with Wnt3a for 4h and lysed with RIPA buffer containing complete protease and phosphatase inhibitor cocktails (Roche). The lysates of control and stimulated cells were centrifuged at 10000 rpm for 10 min at 4˚C. Supernatants were used to measure the GSK3β kinase activity according to the manufacturer`s instruction (GSK3β Assay Kit, #79700, BPS Bioscience). Briefly, 10μg protein was mixed with 25μl of master mix (5μl of 5x Kinase assay buffer + 1μl of 500μM ATP + 5μl of 10x GSK3b substrate peptide + 14μl distilled water) in a 96-well plate. 20 μl of GSK3β enzyme (0.6 ng/μl) was added to each well except blank (20μl of 1x Kinase assay buffer). Plate was incubated at 30°C for 45 minutes, after which 50μl of Kinase-Glo® Max reagent (V6071, Promega) was added to each well and cover with aluminum foil. Following 15 minutes incubation at room temperature, luminescence was measured in a microplate luminometer. The kinase activity is inversely related in luminescence (RLU) reading.

Induction of colitis in mice. Colitis was induced by feeding mice *ad libitum* with 3% DSS (molecular mass, 36–40 kDa; TdB Consultancy) in regular drinking water for 5 days, followed by

normal drinking water until the end of the experiment. Body weight changes, diarrhea and occult bleeding were monitored daily. Diarrhea scoring (0-3) was done as 0 = well-formed pellets, 1 = semiformed stools that did not adhere to the anus, 2 = semiformed stools that adhered to the anus, 3 = liquid stools that adhered to the anus. Bleeding scoring (0-3) was done as 0 = no blood by using hemoccult (Helena Pharmaceuticals), 1 = positive hemoccult, 2 = blood traces in stool visible, 3 = gross rectal bleeding.

Bone marrow-derived macrophages (BMDMs) culture and stimulation. Both femurs and tibias were collected from WT and *Nlrp12*$^{-/-}$ mice. Bone marrow cells from both the femurs and tibias were flushed out and cultured in Iscove`s Modified Dulbecco Medium (IMDM) containing 30% L929 conditioned medium, 10% FBS, 1% Pen/Strep and 1% non-essential amino acids as described previously (8). Differentiated macrophages collected at day 6 were cultured with DMEM medium plus 10% FBS and 1% Pen/Strep in 12-well cell culture plates overnight at 37°C in a CO_2 incubator. Cells were then stimulated with Wnt3a (100 ng/mL) or LPS (1 μg/mL). Following the removal of the culture medium and washing with cold PBS, cells were lysed with RIPA buffer and used for Western blot.

Isolation of small intestine and colon epithelial cells. Both small intestines (SI) and colons were collected from mice, opened longitudinally with fine scissors and washed several times with ice-cold PBS. Tissues were then processed for epithelial cell isolation as described previously (8). Briefly, colons were incubated with PBS supplemented with 1mM DTT on ice for 10 min. Both the colon and small intestine were cut into 5 mm pieces and incubated with RPMI supplemented with 5 mM EDTA, 100 U/ml penicillin, and 100 U/ml streptomycin for 30 min at 37°C on a shaker (250 rpm). The supernatant containing separated crypts was collected into a separate tube and further incubated with RPMI supplemented with 3 mg/mL Dispase (Roche) for 10 min at 37°C on a shaker (250 rpm). The cell suspension was filtered through a 70μm cell strainer and centrifuged

at 1200 rpm. After resuspension of the pellet, immune cells were depleted by negative selection using CD45 MicroBeads (Miltenyi Biotech 130-052-301) following the manufacturer`s instructions. Finally, enriched epithelial cells were collected and used for the experiments. To confirm the purity of the epithelial cells, isolated epithelial cells were incubated with CD16/CD32 (eBioscience 14-0161-82) for 15 min at 4°C and then stained with APC-conjugated rat anti-mouse Ep-CAM (Clone G8.8, Biolegend) for 40 min at 4°C. The purity of the epithelial cells was analyzed by LSR-II and FlowJo software (BD Biosciences, San Jose, CA, USA).

Immune and epithelial cell isolation from mouse colorectal tumors. Colorectal tumor tissues were collected from WT and *Nlrp12*$^{-/-}$ mice at 10-12 weeks post AOM/DSS treatment. Tissues were washed with ice-cold PBS and then processed for immune and epithelial cell isolation. Briefly, tissues were mined with a fine scissor and incubated with lysis buffer (RPMI supplemented with 10% FBS, 0.5 mg/mL collagenase type IV and 10 μg/ mL DNase) for 1h at 37 °C with shaking at 250 rpm. After passing through 18G needle, digested tissues were centrifuged for 4 min at 4°C. Pellets were resuspended in RBC lysis buffer (Sigma R7767-100ML) for 1 min at room temperature and filtered through 70-micron strainer. Cell suspensions were centrifuged and the pellets were resuspended in RPMI medium. Epithelial cells were positively selected by staining with CD326 (EpCAM) MicroBeads and magnetic sorting of Microbeads following the manufacturer`s instructions (Miltenyi Biotec 130-105-958). For immune cell isolation, single cells were mixed with an equal volume of ficoll-paque and centrifuged at 1500 RPM for 20 min at room temperature. The middle layer containing immune cells were collected and washed with RPMI supplemented with 2% FBS. Enriched lymphocyte suspension used for the magnetic sorting of immune cells using CD45 MicroBeads following the manufacturer`s instructions (Miltenyi Biotec 130-052-301). The purity of the epithelial cells and infiltrated immune cells was analyzed by LSR-II and FlowJo software (BD Biosciences, San Jose, CA, USA).

Supplemental References

1. Durkin ME, Qian X, Popescu NC, and Lowy DR. Isolation of Mouse Embryo Fibroblasts. *Bio Protoc.* 2013;3(18).
2. Khan S, Waliullah S, Godfrey V, Khan MAW, Ramachandran RA, Cantarel BL, et al. Dietary simple sugars alter microbial ecology in the gut and promote colitis in mice. *Sci Transl Med.* 2020;12(567).
3. Udden SMN, Peng L, Gan JL, Shelton JM, Malter JS, Hooper LV, et al. NOD2 Suppresses Colorectal Tumorigenesis via Downregulation of the TLR Pathways. *Cell Rep.* 2017;19(13):2756-70.
4. Kozich JJ, Westcott SL, Baxter NT, Highlander SK, and Schloss PD. Development of a dual-index sequencing strategy and curation pipeline for analyzing amplicon sequence data on the MiSeq Illumina sequencing platform. *Appl Environ Microbiol.* 2013;79(17):5112-20.
5. Dobin A, Davis CA, Schlesinger F, Drenkow J, Zaleski C, Jha S, et al. STAR: ultrafast universal RNA-seq aligner. *Bioinformatics.* 2013;29(1):15-21.
6. Li B, and Dewey CN. RSEM: accurate transcript quantification from RNA-Seq data with or without a reference genome. *BMC Bioinformatics.* 2011;12:323.
7. Leng N, Dawson JA, Thomson JA, Ruotti V, Rissman AI, Smits BM, et al. EBSeq: an empirical Bayes hierarchical model for inference in RNA-seq experiments. *Bioinformatics.* 2013;29(8):1035-43.
8. Hu S, Peng L, Kwak YT, Tekippe EM, Pasare C, Malter JS, et al. The DNA Sensor AIM2 Maintains Intestinal Homeostasis via Regulation of Epithelial Antimicrobial Host Defense. *Cell Rep.* 2015;13(9):1922-36.
9. Udden SN, Kwak YT, Godfrey V, Khan MAW, Khan S, Loof N, et al. NLRP12 suppresses hepatocellular carcinoma via downregulation of cJun N-terminal kinase activation in the hepatocyte. *Elife.* 2019;8.

Supplemental Figures

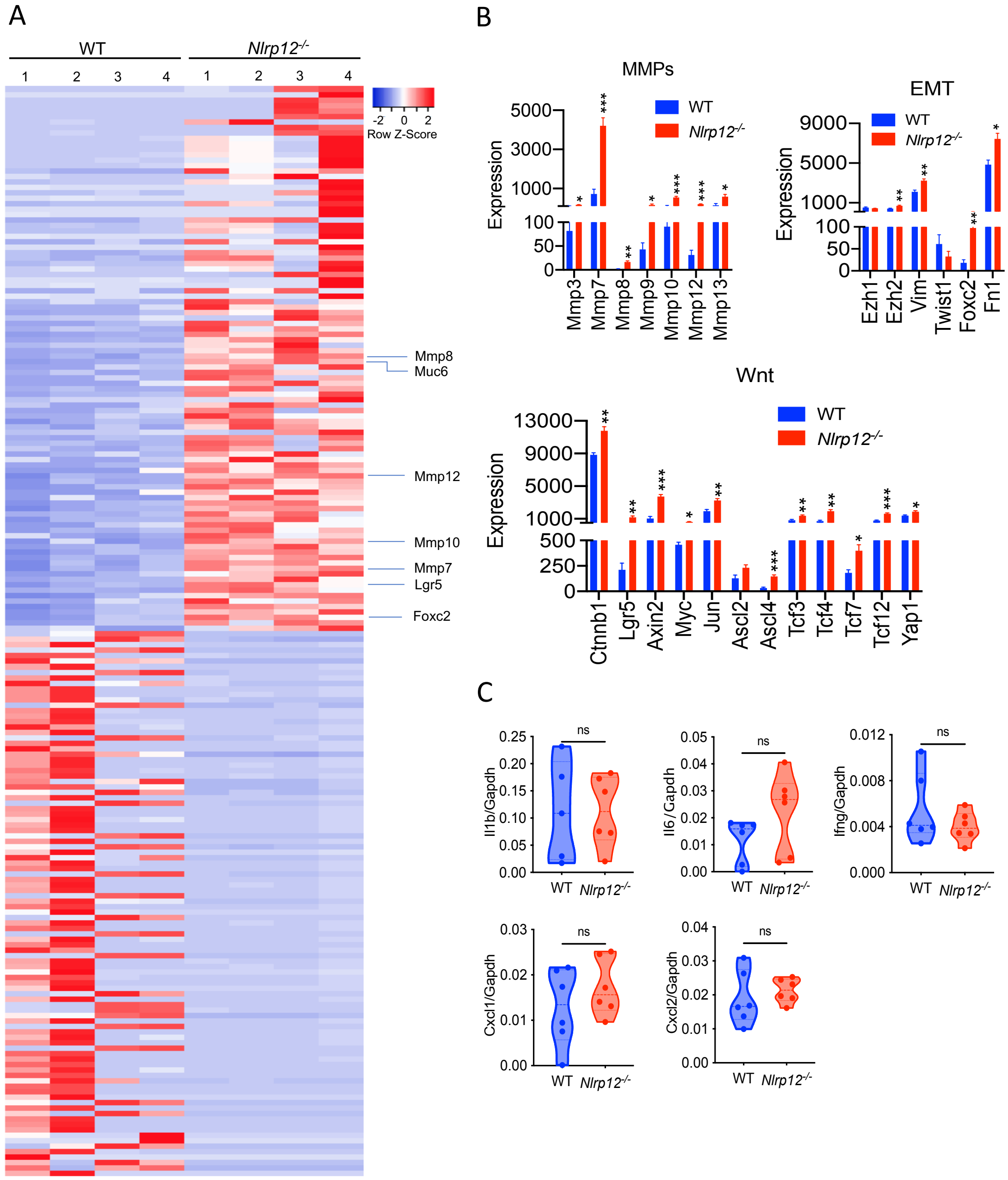

Supplemental Figure 1. NLRP12 suppresses genes involved in proliferation, EMT, and invasion. WT and *Nlrp12*$^{-/-}$ mice were treated with AOM plus 3 cycles of 2.5% DSS. Colorectal tumors were collected at 12 weeks post AOM injection. (A) RNA isolated from WT and *Nlrp12*$^{-/-}$ tumors were analyzed by RNA-seq. Heatmap shows the normalized expression of the top 100 differentially up-and down-regulated genes in WT and *Nlrp12*$^{-/-}$ tumors. (B) Statistical analyses of the expression levels of indicated genes measured by RNA-seq. (C) The expression of indicated genes was measured by real-time RT-PCR. Data represent mean ± SEM; *$p < 0.05$, **$p < 0.01$, ***$p < 0.001$, determined by unpaired Student's *t* test.

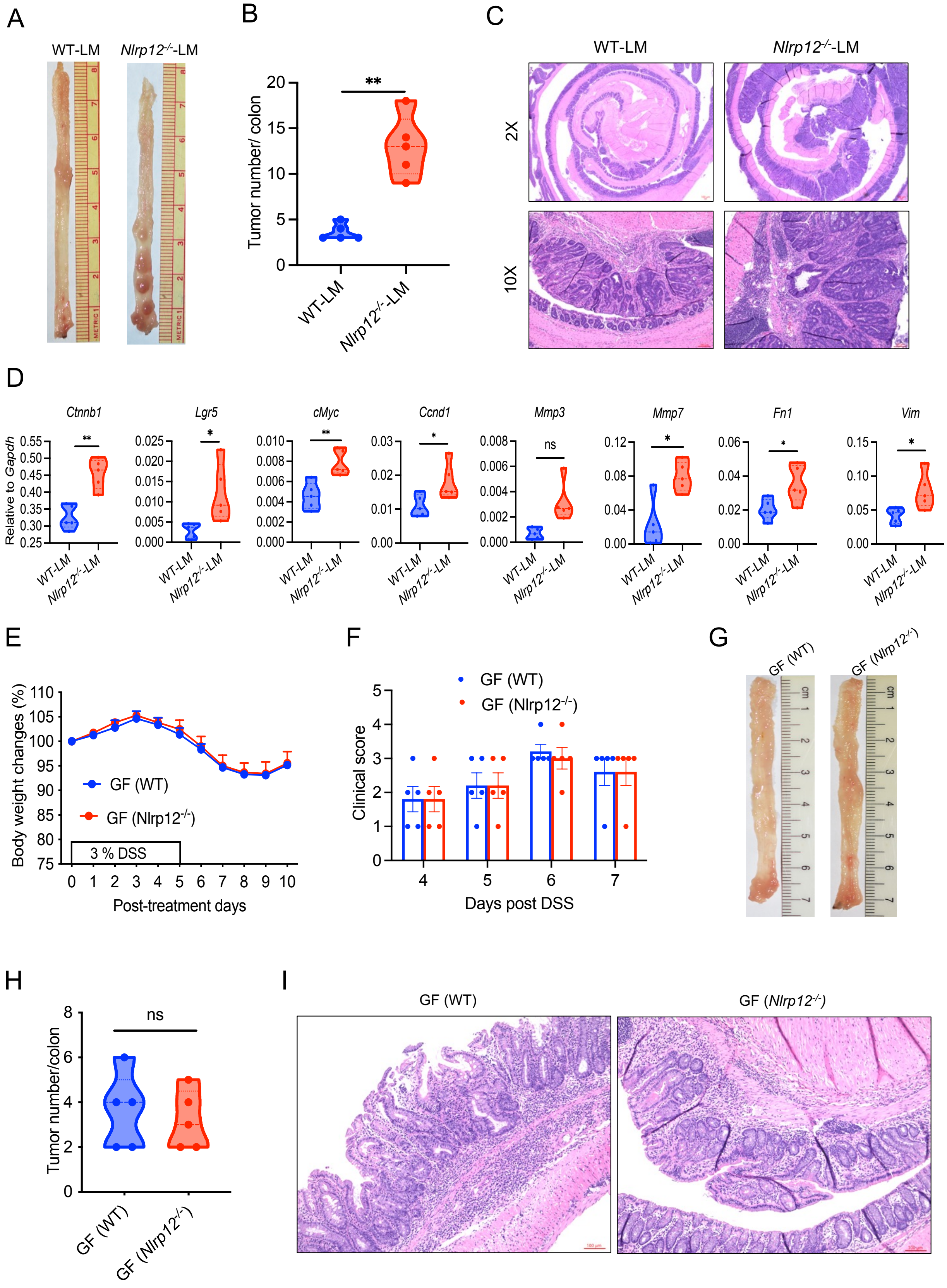
A
WT-LM
Nlrp12-/--LM
B
Tumor number/ colon
WT-LM
Nlrp12-/--LM
C
WT-LM
Nlrp12-/--LM
2X
10X
D
Ctnnb1
Lgr5
cMyc
Ccnd1
Mmp3
Mmp7
Fn1
Vim
Relative to Gapdh
ns
E
Body weight changes (%)
GF (WT)
GF (Nlrp12-/-)
3 % DSS
Post-treatment days
F
Clinical score
Days post DSS
G
GF (WT)
GF (Nlrp12-/-)
H
Tumor number/colon
GF (WT)
GF (Nlrp12-/-)
I
GF (WT)
GF (Nlrp12-/-)

Supplemental Figure 2. Gut microbiota of *Nlrp12*$^{-/-}$ mice are not tumorigenic. (A-D) Littermate WT and *Nlrp12*$^{-/-}$ mice were treated with AOM/DSS regimen. (A-B) 12-week post AOM injection, mice were sacrificed and tumor burden in the colon was counted. (C) Tumor-bearing colon sections were stained with H&E and representative images are shown (scale bar=100μm). (D) RNA isolated from tumor tissues were analyzed for the indicated genes. Data represent mean ± SEM (n=5/group); *p <0.01, **p < 0.001 by unpaired Student's *t* test. (E-I) Fecal homogenates from WT and *Nlrp12*$^{-/-}$ mice were orally gavaged into germ-free (GF) mice. After two weeks of fecal transplantation, fecal recipient mice were treated with AOM plus 3 cycles of DSS (2.5% for 5 days). (E-F) Body weight changes and clinical scores for diarrhea and rectal bleeding were measured during 1st cycle of DSS. (G-H) 10 weeks post AOM, mice were sacrificed, and tumor burden was counted. (I) Histopathological changes in the colons were examined following H&E staining. Data represent mean ± SEM (n=5/group), ns=no significant, determined by unpaired Student's *t* test. All experiments were repeated two times, and data from a representative experiment are presented.

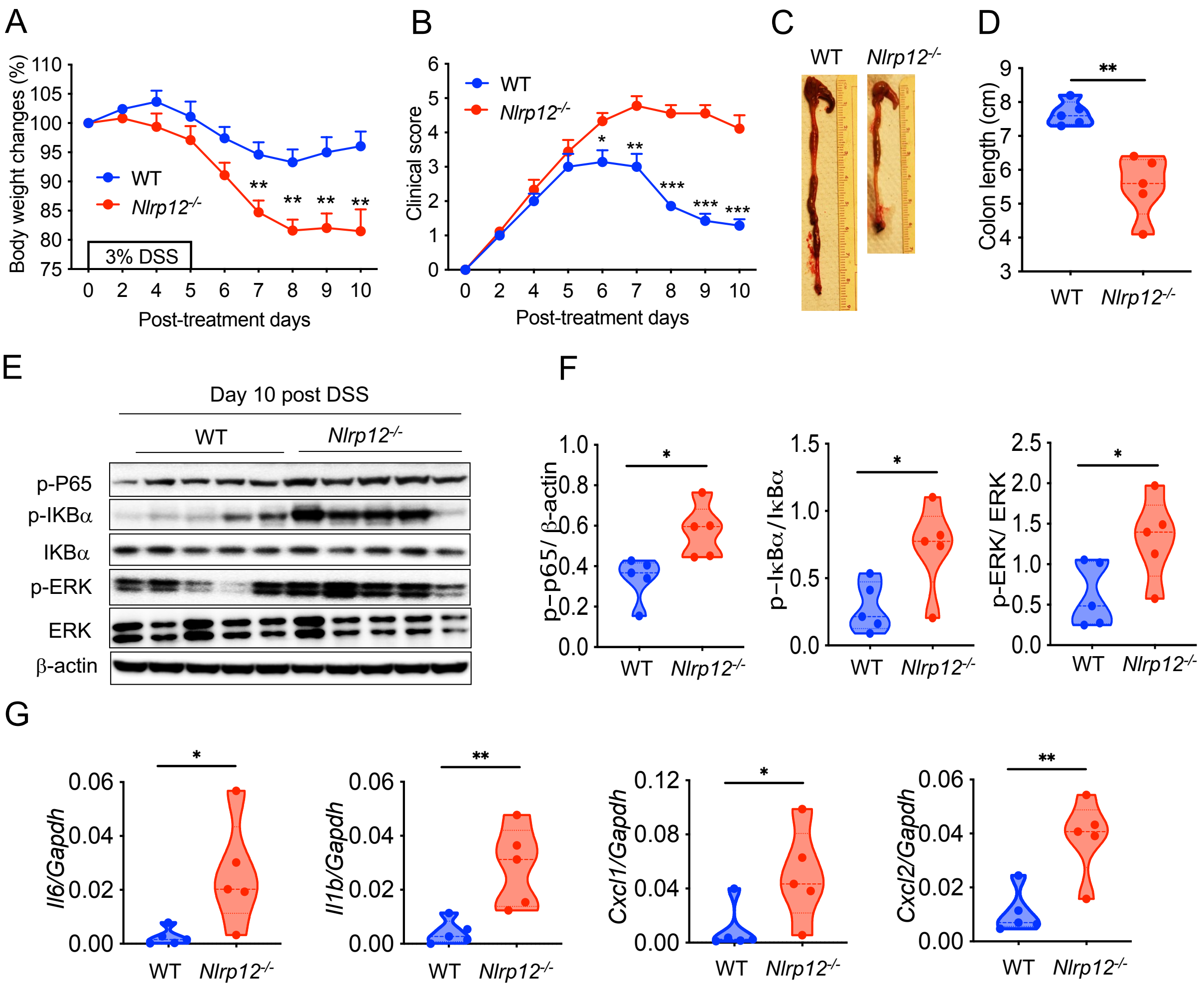

Supplemental Figure 3. NLRP12 dampens NF-κB and ERK activation in the colon during acute colitis. (A-F) WT and *Nlrp12*$^{-/-}$ mice were injected with AOM. Five days post AOM injection, mice were fed with 3% DSS for 5 days. Body weight changes (A), clinical scorings for diarrhea, and rectal bleeding (B) were monitored daily. (C-D) Mice were sacrificed at day 10 and colon lengths were measured. (E) Colon tissue lysates were analyzed for p-P65, p-IκBα, IκBα, p-ERK , ERK and β-actin. (F) Densitometric analysis of band intensities shown in E. (G) RNA isolated from WT and *Nlrp12*$^{-/-}$ colons were used to measure the expression of *Il6*, *Il1b*, *Cxcl1,* and *Cxcl2* by real-time RT-PCR. Data represent mean ± SEM; *$p < 0.05$, **$p < 0.01$, determined by unpaired Student's *t* test. Experiments were repeated three times, and data of a representative experiment are presented.

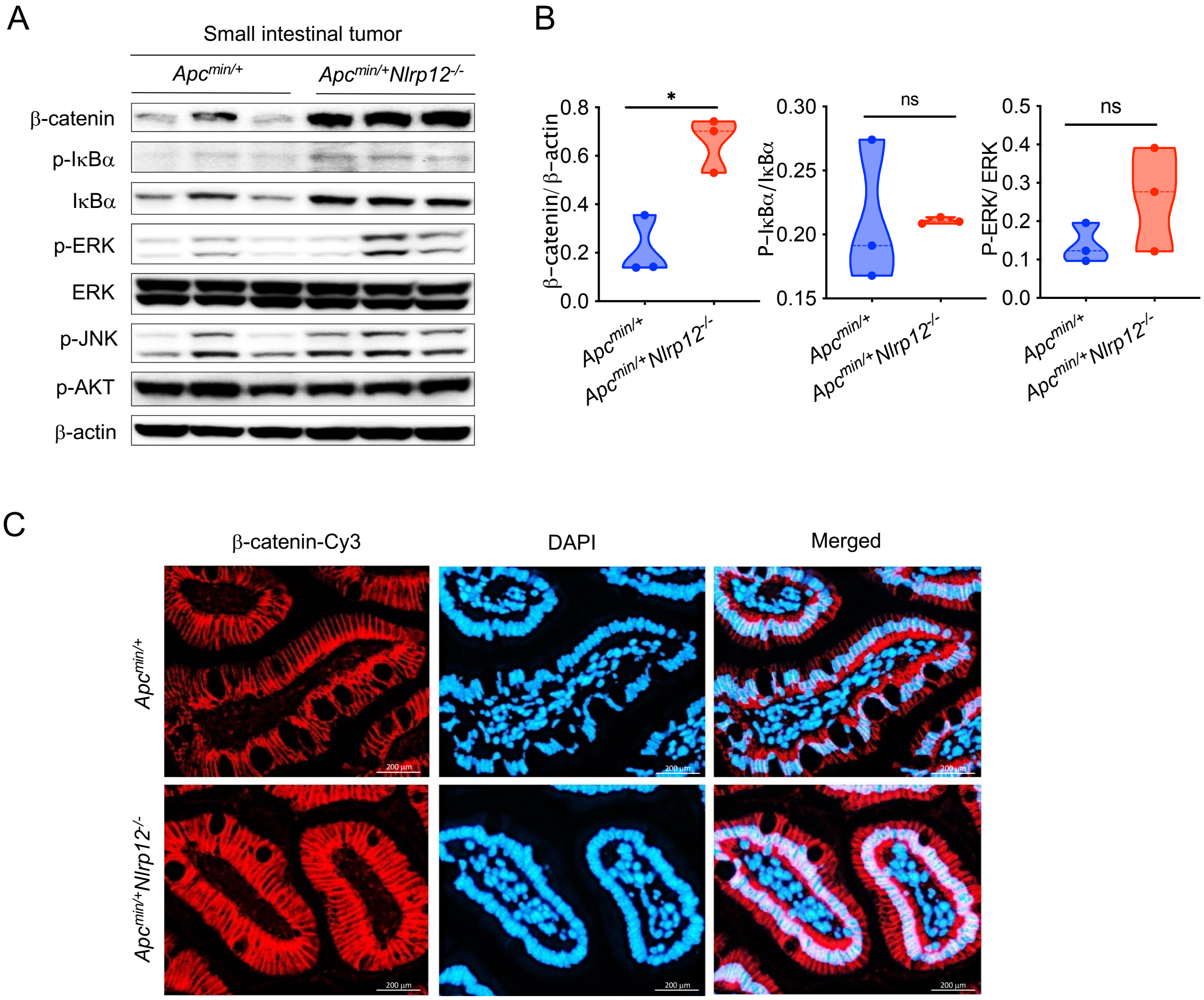

Supplemental Figure 4. Nlrp12 deficiency promotes β-catenin activation in *Apc*$^{min/+}$ mice. *Apc*$^{min/+}$ and *Apc*$^{min/+}$*Nlrp12*$^{-/-}$ mice were sacrificed 5 months after birth. Tumors from the small intestine were processed for Western blotting and analyzed for β-catenin, p-IκBα, IκBα, p-ERK, ERK, p-JNK, p-AKT, and β-actin. (B) Densitometric analyses of p-IκBα, p-ERK and β-catenin reactive bands. Data represent mean ± SD; *p < 0.05, **p < 0.01, determined by unpaired Student's *t* test. (C) Small intestinal tissues were stained for β-catenin (red). The nucleus was stained with DAPI (blue). Experiments were repeated three times, and data from a representative experiment are presented.

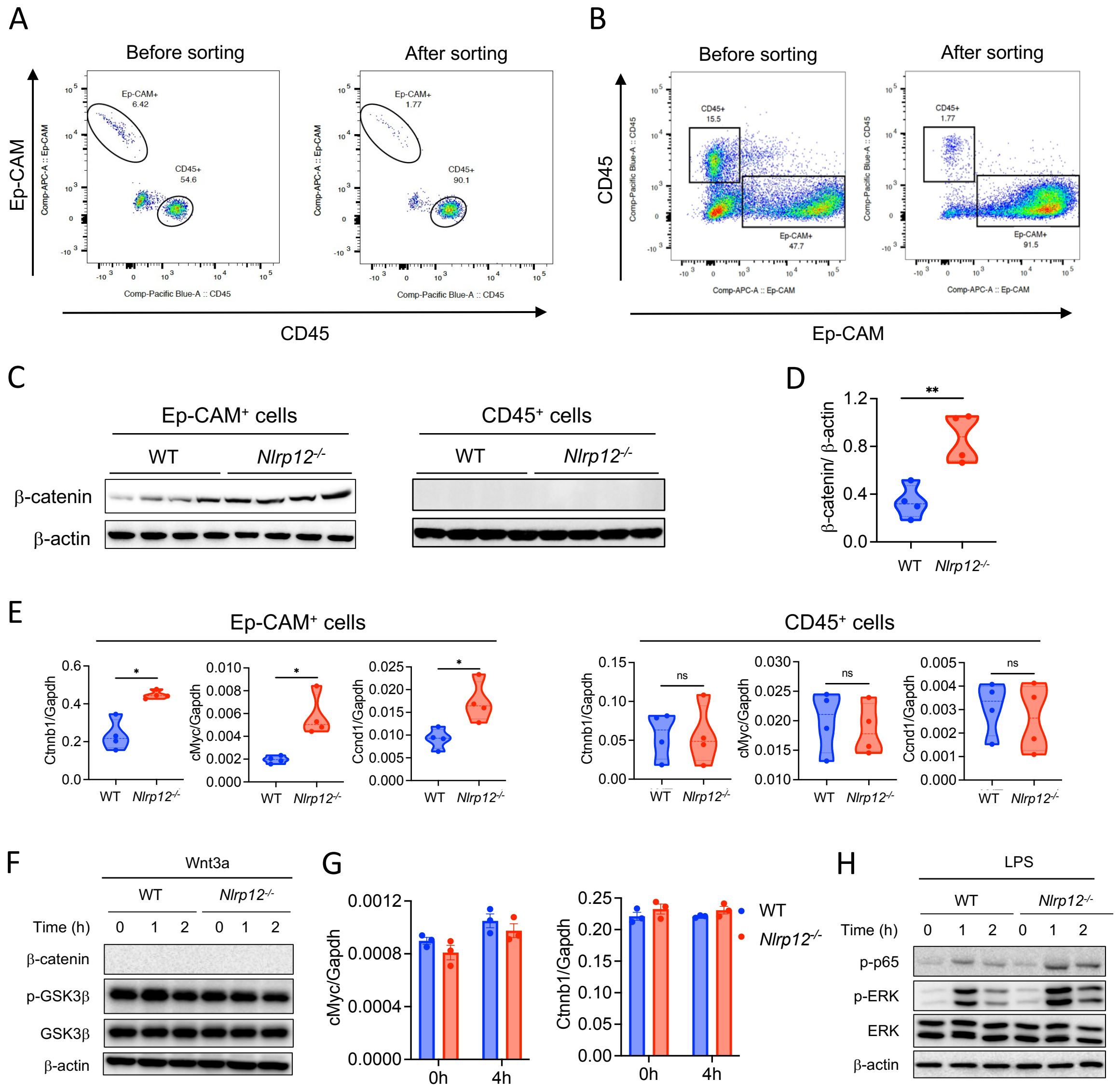

Supplemental Figure 5. Nlrp12 downregulates β-catenin activation in epithelial cells of colorectal tumors. (A-E) Immune cells (CD45+ve cells) and epithelial cells (Ep-CAM+ve cells) were isolated from WT and *Nlrp12*$^{-/-}$ colorectal tumor tissues using magnetic beads. (A-B) The purity of isolated immune cells and epithelial cells were measured by flow cytometry. (C) Ep-CAM^{+} and CD45^{+} cells were measured for β-catenin by Western blotting. (D) Densitometric analysis of β-catenin reactive bands (Ep-CAM^{+} cells). (E) RNA was isolated from Ep-CAM^{+} and CD45^{+} cells and the expression of *Ctnnb1, cMyc, and Ccnd1* was measured by real-time RT-PCR. Data represents mean ± SEM; *p < 0.05, **p < 0.01, determined by unpaired Student's *t* test. Experiments were repeated two times, and data of a representative experiment are presented. (F-G) WT and *Nlrp12*$^{-/-}$ BMDMs were stimulated with Wnt3a (100 ng/mL). (F) The cell lysates were used for measurement of β-catenin, p-GSK3β, GSK3β and β-actin. (G) The expression of *Ctnnb1* and *cMyc* was measured by real-time RT-PCR. (H) BMDMs were stimulated with LPS (1 μg/mL) and the activation of p-P65, p-ERK, ERK and β-actin was measured by Western blotting. Experiments were repeated two times, and data from a representative experiment are presented.

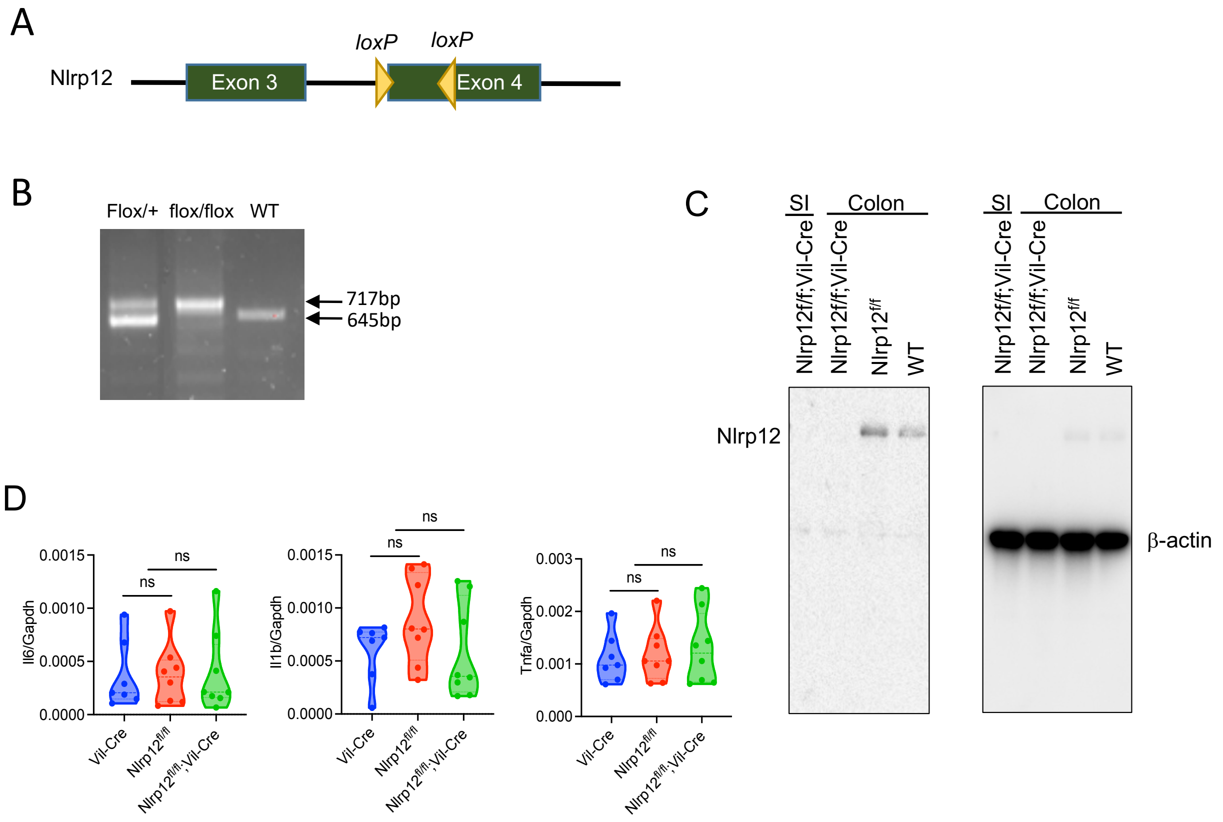

Supplemental Figure 6. Intestinal epithelial cell-specific Nlrp12-KO mice are susceptible to colorectal tumorigenesis. (A) Design of loxP site flanking DNA sequences in exon 4 of *Nlrp12 gene*. (B) Genotyping PCR of founder (F0) Nlrp12$^{flox/flox}$ mice; 717bp band indicates loxP site and 645bp band indicates no loxP site insertion (WT). (C) Epithelial cells were isolated from the small intestine (SI) and colon of respective mice. Western blotting was performed in epithelial cell lysates to measure the expression of Nlrp12. β-actin was used as a loading control. (D) Colorectal tumors were induced in *Nlrp12$^{flox/flox}$*, *Vil-Cre* and *Nlrp12$^{flox/flox}$;Vil-Cre* mice with AOM/DSS treatment. The expression of indicated genes were analyzed by real-time RT-PCR. Data represent mean ± SEM. Statistical analysis was performed by one-way ANOVA. Experiments were repeated at least two times, and data from a representative experiment are presented.

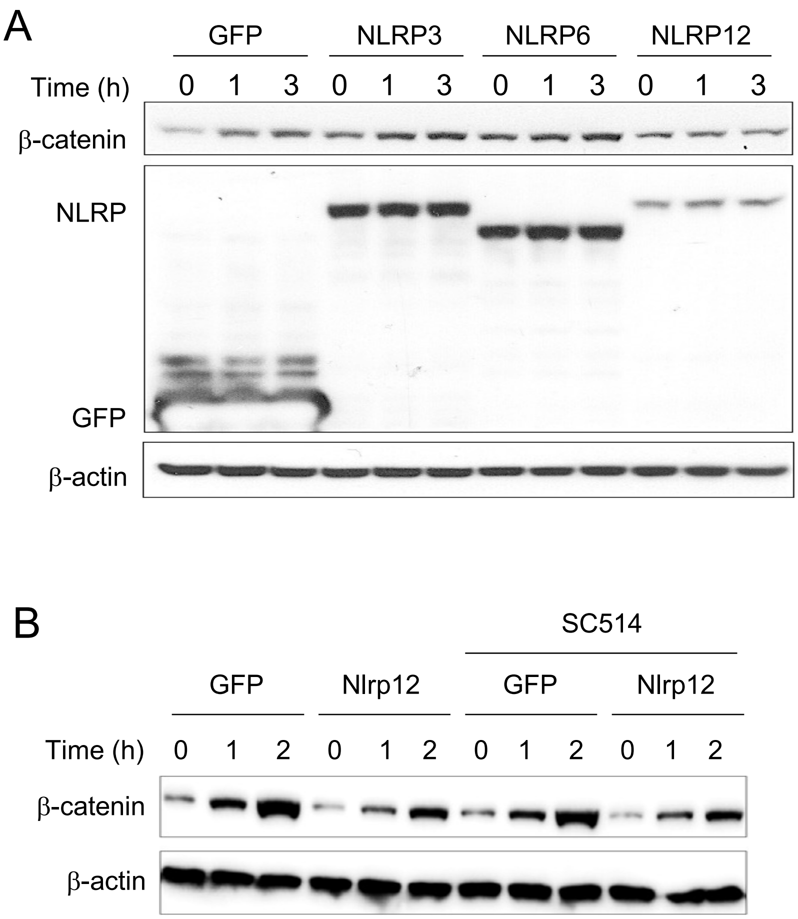

Supplemental Figure 7. NLRP12 suppresses β-catenin activation independent of NF-κB. (A) NLRP3, NLRP6, or NLRP12 were overexpressed in HEK293T cells with transfection of respective flag-tagged plasmids. GFP-plasmid was transfected as a control. Cells were then stimulated with Wnt3a for indicated times and cell lysates were analyzed for β-catenin by Western blotting. The expression of GFP and NLRs was detected by anti-flag antibody. (B) GFP or NLRP12 expressing HEK293T cells were stimulated with Wnt3a in the presence or absence of Sc514, an inhibitor of NF-κB. β-catenin levels were measured by western blotting. Experiments were repeated three times, and data of a representative experiment are presented.

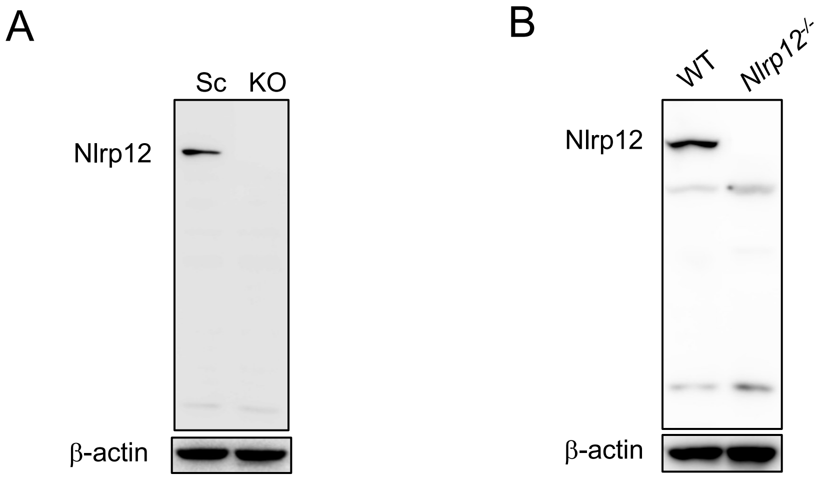

Supplemental Figure 8. Generation of *Nlrp12* KO MC38 cells. (A) Nlrp12 was knocked out in MC38 cells with CRISPR/cas9. Deletion of Nlrp12 in MC38 cells was confirmed by Western blot analysis of Nlrp12. (B) The specificity of Nlrp12 antibody was confirmed by Western blot analysis of colon tissue lysates collected from WT and *Nlrp12*$^{-/-}$ mice.

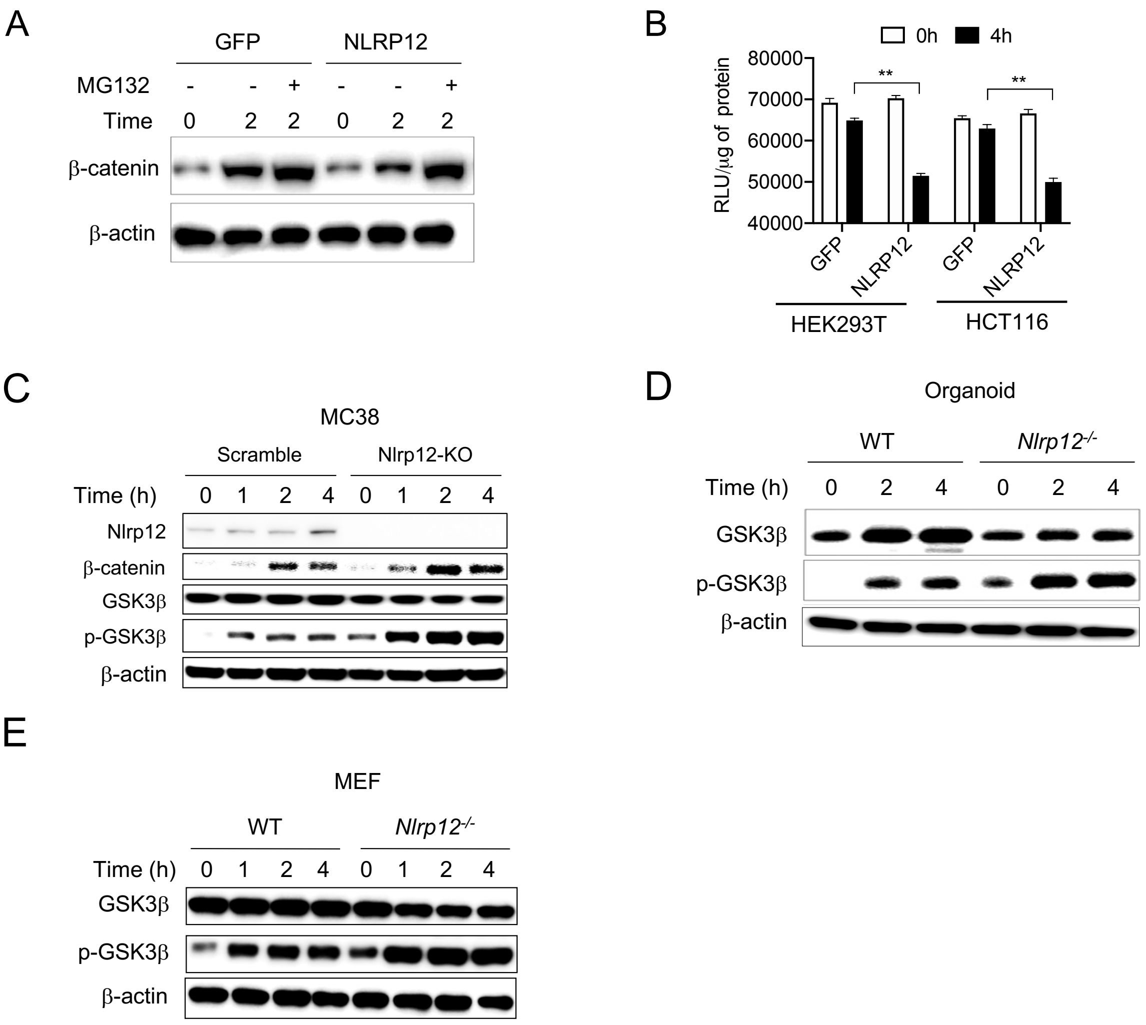

Supplemental Figure 9. NLRP12 inhibits GSK3β phosphorylation and promotes the degradation of β-catenin. (A) GFP or NLRP12 expressing HEK293T cells were stimulated with Wnt3a in the presence or absence of MG132. Cell lysates were analyzed for β-catenin by Western blotting. (B) GFP or NLRP12 expressing HEK293T and HCT116 cells were stimulated with Wnt3a. GSK3β kinase activity in Wnt3a-stimulated HEK293T and HCT116 cell lysates was measured. Kinase activity is inversely related to RLU (relative luminescence unit). (C) Nlrp12 knockout (KO) MC38 cells were generated with CRISPR/Cas9. Scramble or Nlrp12-KO MC38 cells were stimulated with Wnt3a. At indicated times, cells were lysed with RIPA buffer, and the lysates were used to measure Nlrp12, β-catenin, GSK3β, p-GSK3β, and β-actin by Western blotting. (D) Crypts from the small intestine of WT and *Nlrp12*$^{-/-}$ mice were cultured for organoid development. Organoids were stimulated with Wnt3a for 2h and 4h. Cell lysates were measured for GSK3β, p-GSK3β and β-actin by Western blotting. (E) WT and *Nlrp12*$^{-/-}$ MEFs were stimulated with Wnt3a (100 ng/ml). At indicated times, MEFs were lysed with RIPA buffer, and lysates were used to measure GSK3β, p-GSK3β, and β-actin by Western blotting. Experiments were repeated two times, and data from a representative experiment are presented.

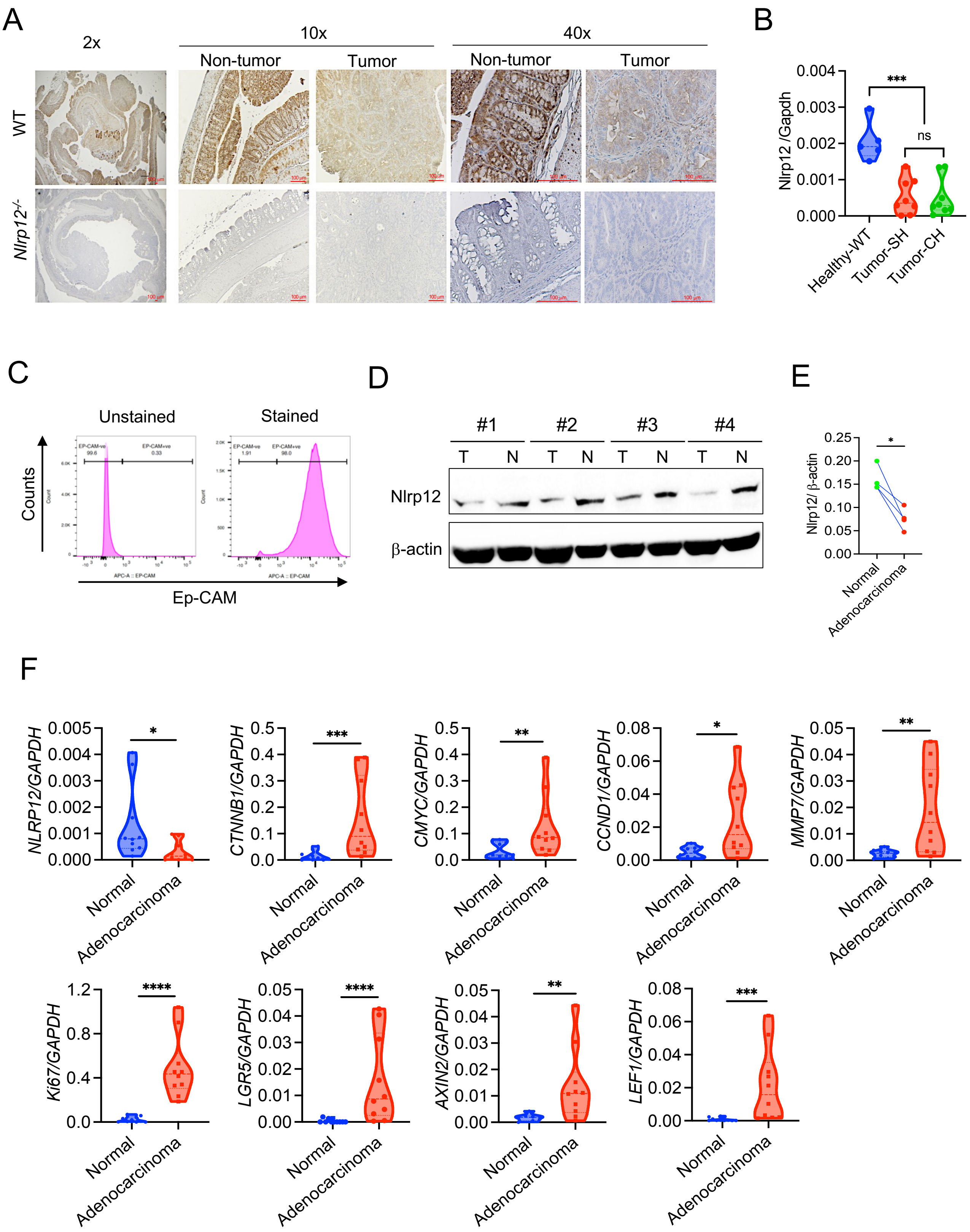
A
2x
10x
40x
Non-tumor
Tumor
Non-tumor
Tumor
WT
Nlrp12-/-
B
Nlrp12 /Gapdh
0.004
0.003
0.002
0.001
0.000
***
ns
Healthy-WT
Tumor-SH
Tumor-CH
C
Unstained
Stained
Counts
Ep-CAM
D
#1
#2
#3
#4
T N T N T N T N
Nlrp12
β-actin
E
Nlrp12/ β-actin
0.25
0.20
0.15
0.10
0.05
0.00
*
Normal
Adenocarcinoma
F
NLRP12/GAPDH
CTNNB1/GAPDH
CMYC/GAPDH
CCND1/GAPDH
MMP7/GAPDH
Ki67/GAPDH
LGR5/GAPDH
AXIN2/GAPDH
LEF1/GAPDH
Normal
Adenocarcinoma

Supplemental Figure 10. The expression of NLRP12 is downregulated in tumor tissues. (A) Tumor-bearing colons of WT and *Nlrp12*$^{-/-}$ mice were immunostained for Nlrp12. (B) WT mice were separately housed or co-housed with *Nlrp12*$^{-/-}$ mice. Colorectal tumors were induced with AOM/DSS treatment. The expression of NLRP12 in the tumor and healthy colons were measured by real-time PCR. Data represent mean ± SEM (n=5-7/group); ***p < 0.001. (C-E) Epithelial cells were isolated from colorectal tumors and adjacent non-tumor tissues from WT mice (n=4) using Ep-CAM MicroBeads. (C) The purity of epithelial cells was measured by flow cytometric analysis of Ep-CAM^{+} cells. (D) Immunoreactive bands of Nlrp12 were measured in isolated epithelial cells by Western blot. (E) Densitometric analysis of Western blot shown in D. (F) Colorectal adenoma and adjacent non-tumor tissue biopsy samples were collected from stage 3 or 4 human colorectal cancer patients. The expression of NLRP12 and Wnt target genes was measured by real-time RT-PCR. Data represent mean ± SEM (n=10/group); **p < 0.01, ***p < 0.001, ****p < 0.0001, analyzed by unpaired Student's *t* test. Experiments were repeated two times, and data of a representative experiment are presented.

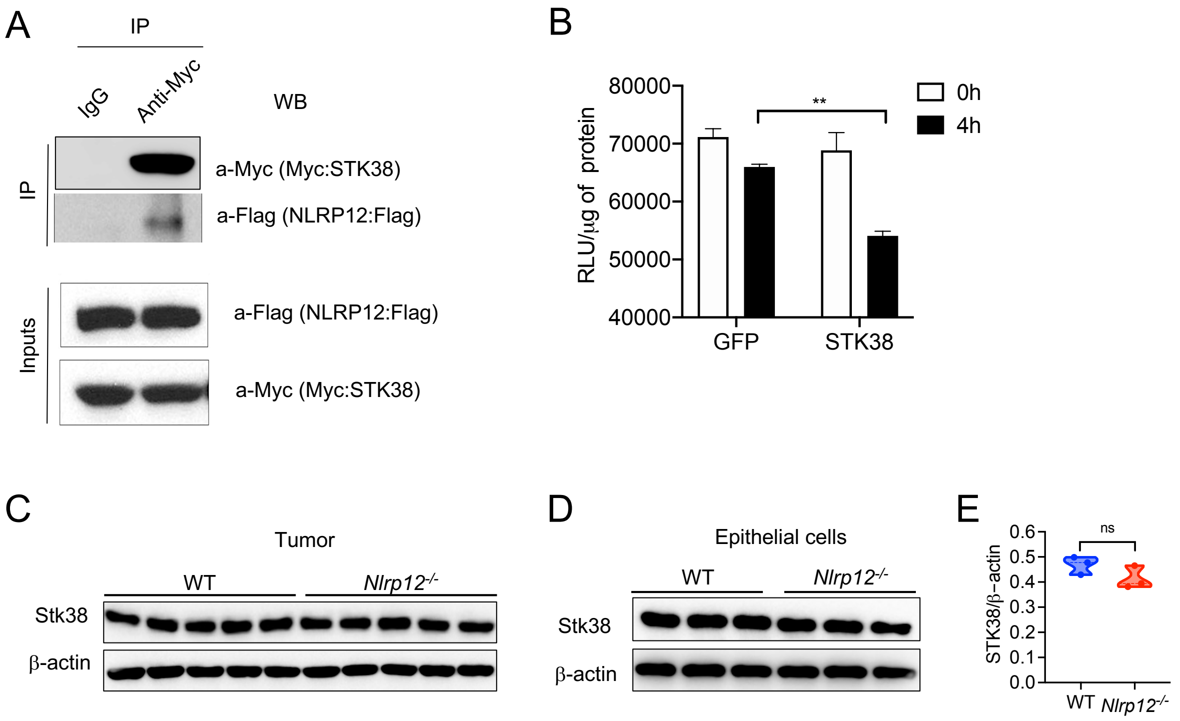

Supplemental Figure 11. NLRP12 regulates the kinase activity of STK38, but not the expression of STK38. (A) Flag-tagged NLRP12 and Myc-tagged STK38 were overexpressed in HEK293T cells. STK38 was pulled down with anti-Myc antibody and immunoblotted with anti-Flag antibody. Inputs were used to measure the expression of NLRP12 and STK38 by western blotting with anti-Flag and anti-Myc antibodies, respectively. (B) STK38-Flag or GFP-Flag were overexpressed in HEK293T cells. Cells were stimulated with Wnt3a for 4 h. Unstimulated and stimulated cell lysates were measured for GSK3β kinase activity, which is inversely related to RLU value. (C-E) Colorectal tumors were induced in WT and *Nlrp12*$^{-/-}$ mice following the AOM/DSS treatment. (C) Immunoreactive bands of Stk38 and β-actin were measured by Western blotting in WT and *Nlrp12*$^{-/-}$ tumor lysates. (D) Epithelial cells were isolated from WT and *Nlrp12*$^{-/-}$ tumors using anti-Ep-CAM MicroBeads. The expression of Stk38 in the tumor epithelium cells were measured by Western blot analysis. (E) Densitometric analysis of Stk38 immunoreactive bands shown in D. Data represent mean ± SD. Experiments were repeated two times, and data of a representative experiment are presented.

Supplemental Tables

Supplemental Table 1. Raw data of RNA-seq analysis showing top 100 up and downregulated genes in *Nlrp12*$^{-/-}$ tumors as shown in Supplementary Figure 1A.

Gene	Name	WT-1	WT-2	WT3	WT-4	KO-1	KO-2	KO-3	KO-4
ENSMUSG00000102364.1	Ighv8-5	0	0	0	0	4.19262067	1.10028098	33.4867861	36.6285412
ENSMUSG00000048830.3	2310057N15Rik	0	0	0	0	0	0	0	71.1438973
ENSMUSG00000046008.7	Pnlip	0	0	0	1.03283582	0	0	698.438681	510.686392
ENSMUSG00000029882.5	2210010C04Rik	0	0	0	2.06567164	0	0	381.749361	230.337173
ENSMUSG00000057163.3	Prss2	0	2.41056737	0.95281607	0	1.04815517	1.10028098	570.232129	409.957903
ENSMUSG00000023140.4	Reg2	0	0	0	1.03283582	0	0	100.460358	108.476834
ENSMUSG00000066108.7	Muc5b	0	1.20528369	0	0	53.4559135	121.030907	0	2.81758009
ENSMUSG00000011463.5	Cpb1	5.173194242	1.20528369	4.76408033	0	0	0	363.57082	308.52502
ENSMUSG00000059654.7	Reg1	0	4.82113475	0	0	2.09631033	0	93.7630011	97.2065132
ENSMUSG00000094818.1	Defa32	0	18.0792553	8.57534459	32.0179104	430.791774	231.059005	28.7029595	1446.82738
ENSMUSG00000074443.1	Defa22	0	24.1056737	20.0091374	50.6089551	663.482221	461.017729	60.276215	2123.0466
ENSMUSG00000095066.1	Defa20	0	18.0792553	15.245057	47.5104477	539.799911	338.88654	48.7950312	1683.50411
ENSMUSG00000074444.4	Defa30	0	18.0792553	31.4429302	80.5611939	705.408427	565.544421	102.373889	2561.1803
ENSMUSG00000085620.2	G630018N14Rik	0	0	0.95281607	0	2.09631033	4.4011239	2.87029595	19.7230606
ENSMUSG00000074447.2	Defa21	0	20.4898227	24.7732177	42.3462686	424.502843	293.77502	36.357082	1776.48425

ENSMUSG00000066755.2	Tnfsf18	1.034638848	0	0	0	11.5297068	3.30084293	4.78382658	8.45274028
ENSMUSG00000098078.1	Gm26992	0.972560517	0	0	0	0	5.03928687	19.1831446	2.15544877
ENSMUSG00000096295.2	Defa2	1.034638848	27.7215248	49.5464354	54.7402984	708.552893	461.017729	69.8438681	2149.10922
ENSMUSG00000055030.1	Sprr2e	1.034638848	0	0	0	0	0	0	26.0626159
ENSMUSG00000076550.3	Igkv4-63	0	1.20528369	5.71689639	1.03283582	9.4333965	9.90252878	63.1465109	107.772439
ENSMUSG00000068078.4	2310034C09Rik	0	6.02641843	2.8584482	1.03283582	1.04815517	0	0	213.431692
ENSMUSG00000074439.4	Defa5	0	9.6422695	7.62252852	6.19701491	142.549103	95.7244448	1.91353063	238.789913
ENSMUSG00000050092.3	Sprr2b	5.173194242	4.82113475	0.95281607	5.1641791	9.4333965	1.10028098	9.56765317	307.11623
ENSMUSG00000042157.2	Sprr2i	4.138555393	0	0	0	1.04815517	0	0	81.7098227
ENSMUSG00000020177.12	9530003J23Rik	0	0	0	1.03283582	5.24077583	6.60168585	7.65412253	1.40879005
ENSMUSG00000063206.4	Defa34	0	1.20528369	0.95281607	3.09850746	20.9631033	19.8050576	0	54.9428118
ENSMUSG00000064213.5	Defa24	2.069277697	121.733652	176.270972	357.361193	2897.10088	1801.15996	340.608453	6403.65515
ENSMUSG00000069722.4	Krtap3-3	5.173194242	1.20528369	0	0	3.1444655	1.10028098	0	104.250463
ENSMUSG00000018727.19	Cpsf4l	0	0	1.90563213	0	11.5297068	3.30084293	12.4379491	4.22637014
ENSMUSG00000069721.2	Krtap3-2	1.034638848	0	0	1.03283582	1.04815517	0	0	30.288986
ENSMUSG00000055826.5	Tescl	1.034638848	0	0	1.03283582	9.4333965	6.60168585	2.87029595	11.9747154
ENSMUSG00000052861.13	Dnah6	1.034638848	0	0.95281607	0	9.4333965	9.90252878	1.91353063	8.45274028
ENSMUSG00000074440.3	Defa3	0	3.61585106	1.90563213	3.09850746	28.3001895	29.7075863	2.87029595	64.8043421
ENSMUSG00000095328.1	Defa-ps6	0	2.41056737	0	0	4.19262067	6.60168585	0	23.9494308

ENSMUSG00000031896.7	Ctrl	5.173194242	4.82113475	6.66971246	5.1641791	7.33708617	0	171.260992	129.608684
ENSMUSG00000050224.3	Krtap13	1.034638848	3.61585106	1.90563213	7.22985073	0	3.30084293	1.91353063	173.985571
ENSMUSG00000056350.7	Krtap13-1	4.138555393	6.02641843	0	0	1.04815517	1.10028098	0	128.904289
ENSMUSG00000108053.1	Gm43890	0	0	1.90563213	0	9.4333965	3.30084293	9.56765317	2.11318507
ENSMUSG00000102308.1	2310046K23Rik	4.138555393	0	0	5.1641791	0	0	0	116.225179
ENSMUSG00000079180.4	Mptx2	0	10.8475532	1.90563213	1.03283582	78.6116375	59.4151727	1.91353063	28.8801959
ENSMUSG00000068323.12	Slc4a5	0	0	0	2.06567164	11.5297068	4.4011239	5.7405919	3.52197512
ENSMUSG00000040809.10	Chil3	0	3.61585106	3.81126426	7.22985073	35.6372757	25.3064624	66.9735722	41.5593064
ENSMUSG00000076594.2	Igkv6-13	1.034638848	1.20528369	3.81126426	26.8537313	6.288931	5.50140488	224.839849	142.287795
ENSMUSG00000081392.1	Gm11668	0	0	0.95281607	2.06567164	13.6260172	8.8022478	3.82706127	8.45274028
ENSMUSG00000112677.1	Gm48868	0	1.20528369	0.95281607	0	10.4815517	2.20056195	7.65412253	4.22637014
ENSMUSG00000030108.14	Slc6a13	0	1.20528369	2.8584482	4.13134328	27.2520343	15.4039337	22.0056023	26.7670109
ENSMUSG00000087149.8	Itih5l-ps	1.034638848	2.41056737	3.81126426	2.06567164	18.866793	28.6073054	40.1841433	15.4966905
ENSMUSG00000085139.1	A730046J19Rik	0	15.6686879	12.3866088	9.29552237	80.7079479	69.3177014	224.839849	13.3835054
ENSMUSG00000056856.12	Jakmip3	1.034638848	2.41056737	3.81126426	3.09850746	18.866793	26.4067434	41.1409086	21.1318507
ENSMUSG00000005800.3	Mmp8	1.034638848	2.41056737	0	3.09850746	14.6741723	9.90252878	22.0056023	19.0186656
ENSMUSG00000048191.15	Muc6	6.20783309	32.5426595	45.7351711	52.6746268	319.687326	297.075863	434.371454	289.506354
ENSMUSG00000079186.3	Gzmc	0	0	1.90563213	1.03283582	5.24077583	8.8022478	2.87029595	11.9747154
ENSMUSG00000096499.2	Ighv1-5	1.034638848	6.02641843	0	3.09850746	35.6372757	35.2089912	18.178541	8.45274028

ENSMUSG00000036500.4	Akp3	0	0	2.8584482	1.03283582	13.6260172	11.0028098	2.87029595	9.1571353
ENSMUSG00000081665.2	Gm15922	0	0	0	4.13134328	5.24077583	9.90252878	15.3082451	7.74834525
ENSMUSG00000025592.17	Dach2	3.103916545	6.02641843	2.8584482	2.06567164	38.7817412	45.11152	9.56765317	31.697776
ENSMUSG00000069515.6	Lyz1	284.5256833	2027.28716	1858.94414	2929.12238	18172.9143	11183.2558	7481.90478	22442.0254
ENSMUSG00000095497.2	Igkv1-122	0	6.02641843	4.76408033	5.1641791	8.38524134	6.60168585	32.5300208	84.5274028
ENSMUSG00000093575.1	Gm20695	2.007199366	0	0	2.68537313	7.89260841	16.6802596	11.5003191	2.64148134
ENSMUSG00000105263.1	Gm42427	0	0	4.76408033	13.4268656	46.1188273	51.7132058	2.87029595	44.3768864
ENSMUSG00000002633.4	Shh	3.103916545	6.02641843	34.3013784	22.722388	224.305206	158.44046	68.8871028	68.3263172
ENSMUSG00000112431.1	Gm10741	0	4.82113475	8.57534459	6.19701491	36.6854308	52.8134868	35.4003167	25.3582208
ENSMUSG00000025644.10	Gm7628	5.173194242	0	6.66971246	2.06567164	19.9149482	39.6101151	27.7461942	18.3142706
ENSMUSG00000094164.2	Ighv2-3	1.034638848	2.41056737	4.76408033	9.29552237	7.33708617	0	48.7950312	75.3702675
ENSMUSG00000049538.14	Adamts16	2.069277697	1.20528369	4.76408033	3.09850746	26.2038792	23.1059005	5.7405919	26.7670109
ENSMUSG00000057400.14	Ces1c	8.277110787	12.0528369	4.76408033	7.22985073	88.045034	75.9193873	11.4811838	59.873577
ENSMUSG00000053054.13	Adh6a	6.20783309	2.41056737	0.95281607	5.1641791	36.6854308	26.4067434	6.69735722	35.9241462
ENSMUSG00000110187.1	Gm45496	0	2.41056737	8.57534459	2.06567164	25.155724	15.4039337	41.1409086	9.86153032
ENSMUSG00000115207.1	Gm49132	3.103916545	0	0	2.06567164	6.288931	16.5042146	7.65412253	5.63516018
ENSMUSG00000038242.12	Aox4	10.34638848	8.43698581	20.0091374	20.6567164	110.056293	64.9165775	134.90391	96.5021182
ENSMUSG00000033860.13	Fgg	0	0	0	6.19701491	12.577862	6.60168585	13.3947144	8.45274028
ENSMUSG00000049723.14	Mmp12	3.103916545	50.6219149	35.2541944	36.1492537	171.897447	174.944675	227.710145	241.607493

ENSMUSG00000025075.14	Habp2	15.51958273	95.2174113	57.1689639	73.3313432	428.695463	498.427282	276.505177	361.354647
ENSMUSG00000025091.3	Pnliprp2	33.10844315	120.528369	412.569356	426.561193	2153.95887	1740.6445	1029.47948	1458.0977
ENSMUSG00000113288.1	Gm47982	0	0	0	5.1641791	8.38524134	4.4011239	13.3947144	7.04395023
ENSMUSG00000074417.9	Gm14548	1.034638848	9.6422695	1.90563213	1.03283582	16.7704827	34.1087102	19.1353063	16.9054806
ENSMUSG00000106086.1	Gm43352	3.103916545	14.4634042	26.6788498	27.8865671	124.730465	115.529502	140.644502	73.9614774
ENSMUSG00000025330.6	Padi4	12.41566618	63.8800354	101.951319	96.0537312	379.43217	448.914638	453.50676	438.133704
ENSMUSG00000039809.10	Gabbr2	1.034638848	1.20528369	0.95281607	2.06567164	11.5297068	6.60168585	11.4811838	3.52197512
ENSMUSG00000020218.11	Wif1	85.87502441	996.769609	1051.90894	992.555222	5073.07101	4711.40314	6795.90405	2714.03402
ENSMUSG00000110666.1	Gm9172	0	0	3.81126426	5.1641791	18.866793	19.8050576	8.61088785	7.74834525
ENSMUSG00000054555.11	Adam12	13.45030503	44.5954964	42.8767229	58.8716417	269.375878	344.387945	133.947144	214.136087
ENSMUSG00000033498.14	Strc	47.59338702	10.8475532	2.8584482	17.5582089	177.138223	143.036527	58.3626843	95.7977231
ENSMUSG00000047562.3	Mmp10	23.79669351	235.030319	28.584482	75.3970148	693.878721	491.825596	428.630862	564.220413
ENSMUSG00000022883.11	Robo1	78.63255247	179.587269	243.920913	131.170149	785.06822	908.832085	1349.99586	712.143368
ENSMUSG00000010175.13	Prox1	80.70183017	626.747517	1080.49342	864.483581	3805.85141	4210.77529	4467.13726	3112.72161
ENSMUSG00000110887.1	Cbx3-ps8	2.069277697	16.8739716	12.3866088	22.722388	81.756103	53.9137678	86.1088785	95.0933281
ENSMUSG00000113211.1	4921525O09Rik	0	16.8739716	5.71689639	17.5582089	70.2263962	59.4151727	66.9735722	38.7417263
ENSMUSG00000018623.9	Mmp7	55.87049781	934.094857	695.555728	1194.99104	4938.90715	3246.92916	3806.9692	4817.35756
ENSMUSG00000098814.2	Igkv19-93	7.242471938	7.23170212	20.9619534	34.083582	53.4559135	83.6213541	108.114481	146.514165
ENSMUSG00000071847.12	Apcdd1	190.3735481	433.902127	397.324299	348.065671	1832.17523	1697.73354	3020.50811	1146.7551

ENSMUSG00000020140.15	Lgr5	21.72741582	232.619752	315.382118	275.767164	1392.99822	1530.49084	1027.56595	745.249934
ENSMUSG00000051323.16	Pcdh19	21.72741582	32.5426595	24.7732177	20.6567164	161.415896	181.546361	130.120083	76.0746625
ENSMUSG00000070873.5	Lilra5	0	1.20528369	5.71689639	9.29552237	15.7223275	22.0056195	24.8758982	26.7670109
ENSMUSG00000005883.15	Spo11	3.103916545	1.20528369	15.245057	8.26268655	64.9856203	20.9053385	18.178541	47.8988616
ENSMUSG00000021986.8	Amer2	0	18.0792553	43.829539	50.6089551	176.090068	169.44327	144.471563	119.042759
ENSMUSG00000042179.5	Pnliprp1	26.90061006	180.792553	478.313665	501.958208	1357.36094	1543.69421	1184.47546	2289.28382
ENSMUSG00000046714.7	Foxc2	3.103916545	9.6422695	34.3013784	25.8208955	122.634155	94.6241639	110.984777	61.282367
ENSMUSG00000085811.1	Cep112it	3.103916545	6.02641843	24.7732177	21.6895522	84.9005685	29.7075863	97.5900623	83.1186127
ENSMUSG00000018930.3	Ccl4	3.103916545	4.82113475	0	0	4.19262067	8.8022478	10.5244185	18.3142706
ENSMUSG00000079440.2	Alpi	6.20783309	36.1585106	129.582985	127.038806	3.1444655	2.20056195	0.95676532	4.93076516
ENSMUSG00000029236.4	Nmu	22.76205466	9.6422695	49.5464354	32.0179104	3.1444655	1.10028098	0	0
ENSMUSG00000045776.3	Lrtm1	41.38555393	98.8332623	6.66971246	4.13134328	2.09631033	0	0	3.52197512
ENSMUSG00000023057.5	Fabp2	136.572328	248.28844	807.035207	1169.17015	9.4333965	9.90252878	23.9191329	45.0812815
ENSMUSG00000096225.7	Lhx8	23.79669351	24.1056737	0	0	1.04815517	0	0	0.70439502
ENSMUSG00000030762.11	Aqp8	1951.328868	485.729326	1001.40968	831.432834	16.7704827	12.1030907	68.8871028	61.282367
ENSMUSG00000030399.2	Ckm	5728.795303	11013.8823	225.817407	192.107462	93.2858099	168.342989	30.6164901	346.562351
ENSMUSG00000020839.16	Tmigd1	15.51958273	97.6279786	532.624181	727.116417	2.09631033	11.0028098	22.0056023	15.4966905
ENSMUSG00000025537.12	Phkg1	77.59791363	145.839326	0.95281607	0	3.1444655	2.20056195	0	2.81758009
ENSMUSG00000085979.1	Gm12055	16.55422157	1.20528369	3.81126426	8.26268655	1.04815517	0	0	0

ENSMUSG00000070385.12	Ampd1	163.472938	262.751844	0	0	2.09631033	0	0	13.3835054
ENSMUSG00000044938.8	Klhl31	175.8886042	286.857517	2.8584482	0	2.09631033	7.70196683	0	7.04395023
ENSMUSG00000111000.1	Gm47108	10.34638848	16.8739716	2.8584482	0	1.04815517	0	0	0
ENSMUSG00000050982.13	Apol10a	57.93977551	640.005638	1569.28806	1367.47462	9.4333965	8.8022478	98.5468276	14.0879005
ENSMUSG00000071540.4	3425401B19Rik	129.329856	210.924645	11.4337928	3.09850746	1.04815517	2.20056195	0.95676532	8.45274028
ENSMUSG00000019787.9	Trdn	196.5813812	355.558688	4.76408033	4.13134328	5.24077583	1.10028098	0.95676532	12.6791104
ENSMUSG00000021622.3	Ckmt2	194.5121035	286.857517	18.1035052	12.3940298	9.4333965	4.4011239	0	4.22637014
ENSMUSG00000019933.7	Mrln	24.83133236	14.4634042	0	2.06567164	0	0	0	1.40879005
ENSMUSG00000020475.3	Pgam2	623.8872256	1295.67996	7.62252852	3.09850746	13.6260172	12.1030907	3.82706127	37.3329362
ENSMUSG00000073406.10	H2-Bl	2.069277697	0	14.292241	12.3940298	0	0	0.95676532	0
ENSMUSG00000002500.15	Rpl3l	180.0271596	282.036383	2.8584482	2.06567164	1.04815517	2.20056195	0	12.6791104
ENSMUSG00000079105.4	C7	121.0527453	54.2377659	0	1.03283582	1.04815517	0	0	4.93076516
ENSMUSG00000004360.9	9330159F19Rik	33.10844315	48.2113475	29.537298	17.5582089	1.04815517	1.10028098	0	2.11318507
ENSMUSG00000047419.5	Cmya5	909.4475477	1691.01301	13.3394249	8.26268655	14.6741723	18.7047766	4.78382658	49.3076516
ENSMUSG00000005628.12	Tmod4	122.0873841	220.566915	0	1.03283582	2.09631033	4.4011239	0	4.93076516
ENSMUSG00000027887.11	Sypl2	177.9578819	241.056737	5.71689639	9.29552237	2.09631033	5.50140488	1.91353063	4.93076516
ENSMUSG00000087090.7	Nctc1	42.42019278	67.4958865	0	0	0	2.20056195	0	1.40879005
ENSMUSG00000095385.3	D630033O11Rik	7.242471938	1.20528369	3.81126426	10.3283582	0	0	0	0.70439502
ENSMUSG00000086845.1	Gm13010	9.311749635	9.6422695	0.95281607	3.09850746	0	0	0	0.70439502

ENSMUSG00000053519.15	Kcnip1	12.4156661 8	6.02641843	46.6879872	26.8537313	0	2.20056195	0	0.70439502
ENSMUSG00000031461.4	Myom2	569.0513666	1084.75532	18.1035052	12.3940298	16.7704827	11.0028098	0.95676532	24.6538258
ENSMUSG00000061728.5	Btnl7-ps	1.034638848	3.61585106	29.537298	22.722388	1.04815517	0	0	0.70439502
ENSMUSG00000030592.18	Ryr1	1299.506394	2316.55525	9.52816065	9.29552237	13.6260172	23.1059005	22.9623676	53.5340217
ENSMUSG00000031097.15	Tnni2	3369.818729	6988.23482	19.0563213	17.5582089	42.9743618	50.6129249	17.2217757	212.727297
ENSMUSG00000023267.10	Gabrr2	10.34638848	32.5426595	0.95281607	3.09850746	0	0	0	1.40879005
ENSMUSG00000027559.5	Car3	3970.9439	8587.64627	10.4809767	36.1492537	11.5297068	60.5154536	13.3947144	300.07228
ENSMUSG00000031972.5	Acta1	10879.22749	20061.947	56.2161479	78.4955223	63.9374652	113.32894	47.8382658	721.300504
ENSMUSG00000062410.14	Hsd3b3	262.7982675	102.449113	744.149347	940.913431	12.577862	9.90252878	19.1353063	20.4274557
ENSMUSG00000020216.13	Jsrp1	132.4337726	237.440886	0	0	3.1444655	0	0.95676532	7.04395023
ENSMUSG00000063730.13	Hsd3b2	70.35544169	21.6951064	213.430799	196.238806	3.1444655	2.20056195	4.78382658	4.93076516
ENSMUSG00000032419.8	Tbx18	24.83133236	71.1117375	0	0	0	0	0	2.81758009
ENSMUSG00000039155.15	Cdh26	12.41566618	2.41056737	0.95281607	9.29552237	0	0	0	0.70439502
ENSMUSG00000022484.7	Hoxc10	44.48947048	89.1909928	1.90563213	1.03283582	1.04815517	0	0	2.81758009
ENSMUSG00000030672.12	Mylpf	2308.279271	3598.97709	1.90563213	5.1641791	19.9149482	15.4039337	11.4811838	121.860339
ENSMUSG00000100642.1	Gm28230	1.686461323	4.56802517	50.4230262	55.9177312	1.04815517	0	0	2.09909717
ENSMUSG00000017300.9	Tnnc2	4637.251318	8103.12223	0	2.06567164	25.155724	46.211801	0.95676532	283.871194
ENSMUSG00000055775.16	Myh8	12178.73388	25611.0731	0	0	125.77862	228.858443	1.91353063	695.942283
ENSMUSG00000051747.14	Ttn	2436.574488	4660.83202	38.1126426	43.3791044	47.1669825	33.0084293	11.4811838	106.363648

ENSMUSG00000087339.1	Gm11586	0	1.20528369	12.3866088	13.4268656	0	0	0	0.70439502
ENSMUSG00000015843.10	Rxrg	14.48494388	28.9268085	5.71689639	4.13134328	0	0	0	1.40879005
ENSMUSG00000020722.5	Cacng1	180.0271596	253.109574	0	0	1.04815517	2.20056195	0	8.45274028
ENSMUSG00000027380.10	Acoxl	14.48494388	101.24383	1.90563213	0	1.04815517	1.10028098	0.95676532	0
ENSMUSG00000021850.14	ccdc198	0	4.82113475	40.9710908	25.8208955	1.04815517	0	0	0.70439502
ENSMUSG00000062077.14	Trim54	182.0964373	359.174539	2.8584482	3.09850746	3.1444655	5.50140488	0	4.93076516
ENSMUSG00000027868.11	Tbx15	126.2259395	161.508014	0.95281607	0	1.04815517	1.10028098	0	4.93076516
ENSMUSG00000054630.7	Ugt2b5	0	3.61585106	109.573848	127.038806	0	1.10028098	1.91353063	2.81758009
ENSMUSG00000001656.3	Hoxc11	15.51958273	40.9796454	0	3.09850746	0	0	0	1.40879005
ENSMUSG00000024471.12	Myot	378.6778185	566.483333	0.95281607	2.06567164	5.24077583	4.4011239	1.91353063	10.5659253
ENSMUSG00000063821.6	Dupd1	19.65813812	13.2581206	0	0	0	0	0	0.70439502
ENSMUSG00000110815.1	Gm47345	1.034638848	1.20528369	15.245057	15.4925373	0	0	0	0.70439502
ENSMUSG00000027022.13	Xirp2	521.4579796	1075.11305	7.62252852	6.19701491	13.6260172	3.30084293	0	16.9054806
ENSMUSG00000061723.18	Tnnt3	5229.06474	11664.7355	2.8584482	1.03283582	26.2038792	62.7160156	0.95676532	258.512973
ENSMUSG00000068373.15	D430041D05Rik	5.173194242	32.5426595	0	0	0	0	0	0.70439502
ENSMUSG00000030996.8	Art1	82.77110787	94.0121276	4.76408033	0	0	0	0	3.52197512
ENSMUSG00000030730.12	Atp2a1	4767.615813	9208.36737	0	2.06567164	35.6372757	69.3177014	0.95676532	162.010855
ENSMUSG00000038670.11	Mybpc2	986.0108225	2879.42273	16.1978731	9.29552237	11.5297068	22.0056195	1.91353063	37.3329362
ENSMUSG00000073402.11	Gm8909	1.034638848	6.02641843	66.6971246	37.1820895	1.04815517	0	0.95676532	0

ENSMUSG00000016327.9	Atp1b4	12.41566618	28.9268085	0	0	0	0	0	0.70439502
ENSMUSG00000067231.4	Cyp2c65	68.28616399	31.3373759	1057.62583	1003.91642	8.38524134	2.20056195	11.4811838	16.2010855
ENSMUSG00000111283.1	E230034D01Rik	1.034638848	0	31.4429302	29.9522388	1.04815517	0	0	0
ENSMUSG00000073403.11	Gm10499	6.20783309	4.82113475	15.245057	16.5253731	0	0	0	0.70439502
ENSMUSG00000026100.6	Mstn	41.38555393	87.9857091	0	0	0	0	0	2.11318507
ENSMUSG00000013653.2	1810065E05Rik	468.6913983	509.835	7680.6503	8491.9761	24.1075688	29.7075863	157.866277	66.2131322
ENSMUSG00000068697.7	Myoz1	594.9173378	1046.18624	0	0	1.04815517	11.0028098	0	14.0879005
ENSMUSG00000042540.12	Acot5	13.45030503	31.3373759	0	3.09850746	0	0	0	0.70439502
ENSMUSG00000044951.15	Mylk4	287.6295998	460.418368	1.90563213	0	2.09631033	1.10028098	0	7.74834525
ENSMUSG00000054128.16	H2-T3	0	14.4634042	242.015281	265.438806	0	0	3.82706127	3.52197512
ENSMUSG00000079278.1	Tmem233	77.59791363	95.2174113	0	0	0	0	0.95676532	1.40879005
ENSMUSG00000061816.15	Myl1	2169.637665	3949.71464	2.8584482	1.03283582	5.24077583	11.0028098	0	68.3263172
ENSMUSG00000030433.15	Sbk2	256.5904344	296.499787	0.95281607	1.03283582	2.09631033	2.20056195	1.91353063	1.40879005
ENSMUSG00000040705.2	A930016O22Rik	18.62349927	32.5426595	0	4.13134328	0	0	0	0.70439502
ENSMUSG00000057003.12	Myh4	105.5331625	115.707234	0	0	1.04815517	1.10028098	0	0.70439502
ENSMUSG00000116378.1	Gcat	15.41611884	55.563578	29.3181503	0	0	1.25432031	0	0
ENSMUSG00000040113.14	Mettl11b	37.24699854	42.184929	0	0	0	0	0	0.70439502
ENSMUSG00000049173.7	Myoz3	48.62802587	51.8271985	0	0	0	0	0	0.70439502
ENSMUSG00000006457.3	Actn3	828.7457175	2517.83762	0	7.22985073	1.04815517	15.4039337	2.87029595	1.40879005

ENSMUSG00000025002.5	Cyp2c55	1333.649476	127.760071	5110.90537	3700.65074	5.24077583	5.50140488	11.4811838	32.4021711
ENSMUSG00000035923.4	Myf6	65.18224745	87.9857091	0	0	0	0	0	0.70439502
ENSMUSG00000026614.6	Slc30a10	270.0407394	1.20528369	639.33958	375.952238	1.04815517	2.20056195	0	2.81758009
ENSMUSG00000005716.16	Pvalb	344.5347365	532.73539	0	0	1.04815517	0	0	2.81758009
ENSMUSG00000033044.12	Dhrs7c	63.11296975	107.270248	0.95281607	1.03283582	0	0	0	0.70439502
ENSMUSG00000056328.14	Myh1	10611.25603	20987.6048	0	0	5.24077583	13.2033717	1.91353063	65.5087371
ENSMUSG00000102049.1	Zbed6	8.959972427	0	0	50.6502686	0	0	0	0
ENSMUSG00000094478.1	Igkv3-3	0	0	0	73.3313432	0	0	0	0
ENSMUSG00000096580.1	Igkv1-132	0	10.8475532	40.0182747	23.7552238	0	0	0	0
ENSMUSG00000028396.5	2310002L09Rik	35.17772084	51.8271985	0.95281607	0	0	0	0	0
ENSMUSG00000109941.1	Exosc6	113.0239478	0	0	0	0	0	0	0
ENSMUSG00000097615.1	Gm2061	50.69730357	2.41056737	103.856951	52.6746268	0	0	0	0
ENSMUSG00000090015.8	Gm15446	79.66719132	0	60.0274121	82.6268655	0	0	0	0
ENSMUSG00000085348.1	Myhas	104.4985237	232.619752	0	0	0	0	0	0

Supplemental Table 2. List of primers for measurement of mouse (m) and human (h) mRNA by real-time qPCR.

Gene Name	Forward	Reverse
mIl1b	GCCTCGTGCTGTCGGACCCATA	TGCAGGGTGGGTGTGCCGTCTT
mIl6	CAA GAA AGA CAA AGC CAG AGT C	GAA ATT GGG GTA GGA AGG AC
mTNFa	TCCCAGGTTCTCTTCAAGGGA	GGTGAGGAGCACGTAGTCGG
mIFNg	GAAAGACAATCAGGCCATCA	TTGCTGTTGCTGAAGAAGGT
mCxcl1	TGAGCTGCGCTGTCAGTGCCT	AGAAGCCAGCGTTCACCAGA
mCxcl2	CAA GAA CAT CCA GAG CTT GAG TGT	GCC CTT GAG AGT GGC TAT GAC TT
mKi67	AGAAGTCCAGGTCTACAG	TCGTTGCTATTGCTAAGG
mCdh2	CAATGACGTCCACCCTGTTCT	CAATGACGTCCACCCTGTTCT
mFn1	CCCAGACTTATGGTGGCAATT	ATATTCCGACTCGAGTCTGA
mVim	CCGTTCAAGGTCAAGACGTGCCA	AGGAGGCCGAAAGCACCCTGC
mEzh2	CAGGCTGGGGCATCTTTATC	ACGAATTTTGTTGCCCTTTC
mZeb2	CAGATCAGCACCAAATGCTAAC	ACACTCCGTGCACTTGAACTT
mFoxc2	GCAACCCAACAGCAAACTTTC	GACGGCGTAGCTCGATAGG
mMmp3	ACCAACCTATTCCTGGTTGCTGCT	ATGGAAACGGGACAAGTCTGTGGA
mMmp7	ACCAACCTATTCCTGGTTGCTGCT	ATGGAAACGGGACAAGTCTGTGGA
mMmp8	AATCCTTGCCCATGCCTTTCAACC	CCAAATTCATGAGCAGCCACGAGA
mMmp10	GACCCCAGACAAATGTGATCCT	TTCAGGCTCGGGATTCCA
mMmp12	TAGAAGCAACTGGGCAACTGGACA	ACCGCTTCATCCATCTTGACCTCT
mMmp13	TTCTGGTCTTCTGGCACACGCTTT	CCAAGCTCATGGGCAGCAACAATA
mCtnnb1	AAG GGC AAG GTT CGA ATC AA	AGC CGA GAT GGC CCA GAA T
mLgr5	CGGGACCTTGAAGATTTCCT	GATTCGGATCAGCCAGCTAC
mMyc	CCTTTGGGCGTTGGAAACC	TCCTCGTCGCAGATGAAATAGG
mAxin2	GCAGCTCAGCAAAAAGGGAAAT	TACATGGGGAGCACTGTCTCGT
mCcnd1	TGCCATCCATGCGGAAA	AGCGGGAAGAACTCCTCTTC
mYap1	TACATAAACCATAAGAACAAGACCACA	GCTTCACTGGAGCACTCTGA
mNlrp12	CCT CTT TGA GCC AGA CGA AG	GCC CAG TCC AAC ATC ACT TT
mGapdh	TGG CAA AGT GGA GAT TGT TGC C	AAG ATG GTG ATG GGC TTC CCG

hNLRP12	AAATGCACTGGAGGATTTGG	CAGGCTCTGGTTCACACTGA
hCTNNB1	CAGAAGCTATTGAAGCTGAGG	TTCCATCATGGGGTCCATAC
hAXIN2	ACTGCCCACACGATAAGGAG	CTGGCTATGTCTTTGGACCA
hLEF1	CTTTATCCAGGCTGGTCTGC	TCGTTTTCCACCATGTTTCA
hMYC	TGAGGAGACACCGCCCAC	CAACATCGATTTCTTCCTCATCTTC
hCCND1	AAGCTCAAGTGGAACCT	AGGAAGTTGTTGGGGC
hMMP7	TGAGCTACAGTGGGAACAGG	TCATCGAAGTGAGCATCTCC
hKI67	GAGGTGTGCAGAAAATCCAAA	CTGTCCCTATGACTTCTGGTTGT
hLGR5	CTTCCAACCTCAGCGTCTTC	TTTCCCGCAAGACGTAACTC
hGAPDH	TCTGGTAAAGTGGATATTGTTG	GATGGTGATGGGATTTCC

Supplemental Table 3. Patients information of colorectal high-grade adenocarcinoma specimens.

Case	Gender	Age	Path Dx	Path comments	Tumor Size (cm)	Tumor Grade	IOR Treatment	Anatomy Site
1	Male	68	Poorly differentiated signet ring cell adenocarcinoma with mucinous features	Histologic Grade: High-grade, poorly differentiated	4.7	High	No	Colon, NOS
2	Male	80	Adenocarcinoma		13.5	4	No	Sigmoid colon
3	Male	69	Adenocarcinoma	Histologic Grade: High-grade	7.5	4	No	Rectum, NOS
4	Female	66	Adenocarcinoma	Histologic Grade: poorly differentiated	4.5	High	Yes	Colon, NOS
5	Female	67	Poorly differentiated adenocarcinoma with neuroendocrine features		2	High	No	Rectum, NOS
6	Female	44	Mucinous adenocarcinoma-Signet-ring cell carcinoma		9	3	No	Colon, NOS
7	Female	71	Adenocarcinoma	Histologic Grade: poorly differentiated with signet-ring cell features	3.2	High	No	Colon, NOS
8	Female	58	Adenocarcinoma with focal medullary features (40%)		10.8	3	No	Colon, NOS
9	Female	54	Adenocarcinoma	Invasive adenocarcinoma, poorly-differentiated with focal mucinous features, arising from tubular adenoma with high-grade dysplasia	4.2	3	No	Colon, NOS
10	Male	51	Adenocarcinoma		3.8	3	No	Colon, NOS
